# Patient-reported outcome measures for pain in autosomal dominant polycystic kidney disease: A systematic review

**DOI:** 10.1371/journal.pone.0252479

**Published:** 2021-05-27

**Authors:** Patrizia Natale, Elyssa Hannan, Bénédicte Sautenet, Angela Ju, Ronald D. Perrone, Eva Burnette, Niek Casteleijn, Arlene Chapman, Sarah Eastty, Ron Gansevoort, Marie Hogan, Shigeo Horie, Bertrand Knebelmann, Richard Lee, Reem A. Mustafa, Richard Sandford, Amanda Baumgart, Allison Tong, Giovanni F. M. Strippoli, Jonathan C. Craig, Gopala K. Rangan, Yeoungjee Cho

**Affiliations:** 1 Sydney School of Public Health, The University of Sydney, Sydney, NSW, Australia; 2 Centre for Kidney Research, The Children’s Hospital at Westmead, Westmead, NSW, Australia; 3 Department of Emergency and Organ Transplantation, University of Bari, Bari, Italy; 4 Service de Néphrologie-Hypertension, Dialyses, Transplantation Rénale, Hôpital de Tours, Tours, France; 5 Université de Tours, Université de Nantes, INSERM, SPHERE U1246, Tours, France; 6 Medicine, Nephrology, Clinical and Translational Research Center, Tufts Medical Center and Tufts University School of Medicine, Boston, Massachusetts, United States of America; 7 London, United Kingdom; 8 Department of Urology, University Medical Centre Groningen, University of Groningen, Groningen, The Netherlands; 9 Department of Nephrology, The University of Chicago, Chicago, Illinois, United States of America; 10 Division of Nephrology & Hypertension, Department of Internal Medicine Mayo Clinic, Rochester, Minnesota, United States of America; 11 Department of Urology, Juntendo University, Tokyo, Japan; 12 Université de Paris APHP, Hôpital Necker, Service de Néphrologie, Paris, France; 13 Armidale, NSW, Australia; 14 Division of Nephrology and Hypertension, Department of Internal Medicine, University of Kansas Medical Centre, Lawrence, Kansas, United States of America; 15 Academic Department of Medical Genetics, University of Cambridge, Cambridge, United Kingdom; 16 College of Medicine and Public Health, Flinders University, Adelaide, SA, Australia; 17 Centre for Transplant and Renal Research, Westmead Institute for Medical Research, The University of Sydney, Sydney, NSW, Australia; 18 Department of Medicine, Westmead Hospital, Western Sydney Local Health District, Sydney, NSW, Australia; 19 Australasian Kidney Trials Network, University of Queensland, Brisbane, QLD, Australia; 20 Translational Research Institute, Brisbane, QLD, Australia; 21 Department of Nephrology, Princess Alexandra Hospital, Brisbane, QLD, Australia; Iranian Institute for Health Sciences Research, ISLAMIC REPUBLIC OF IRAN

## Abstract

Pain is a common symptom in people with autosomal dominant polycystic kidney disease (ADPKD), but it is assessed and reported inconsistently in research, and the validity of the measures remain uncertain. The aim of this study was to identify the characteristics, content, and psychometric properties of measures for pain used in ADPKD. We conducted a systematic review including all trials and observational studies that reported pain in people with ADPKD. Items from all measures were categorized into content and measurement dimensions of pain. We assessed the general characteristics and psychometric properties of all measures. 118 studies, we identified 26 measures: 12 (46%) measures were developed for a non-ADPKD population, 1 (4%) for chronic kidney disease, 2 (8%) for polycystic liver disease and 11 (42%) specifically for ADPKD. Ten anatomical sites were included, with the lower back the most common (10 measures [39%]), four measurement dimensions (intensity (23 [88%]), frequency (3 [12%]), temporality (2 [8%]), and sensory (21 [81%]), two pain types, nociceptive including visceral (15 [58%]) and somatic (5 [20%]), and neuropathic (2 [8%]), and twelve impact dimensions, where the most frequent was work (5 [31%]). The validation data for the measures were variable and only the ADPKD Impact Scale reported all psychometric domains. The measures for pain in ADPKD varied in terms of content and length, and most had not been validated in ADPKD. A standardized psychometrically robust measure that captures patient-important dimensions of pain is needed to evaluate and manage this debilitating complication of ADPKD.

## Introduction

Pain is a debilitating symptom that is experienced by more than 60% of people with autosomal dominant polycystic kidney disease (ADPKD) by the age of 40 years old [1]. The progressive growth of cysts in the kidneys and cyst complications including infection and rupture [2] can cause extreme acute or persistent chronic pain [3] if pain lasts for longer than 4–6 weeks [4]. ADPKD-related pain impacts on sleep quality and physical activity, impairs well-being and overall quality of life due to its recurrent nature and severity [5], necessitating regular analgesics in up to 30% of people with ADPKD [6, 7]. People with ADPKD report pain in a range of sites including the lower back (71%), abdomen (61%), head (49%), and chest (30%) [8], and the onset of pain can be sudden and unpredictable [9]. Cyst-related pain is often persistent and aggravated by standing and walking and the source of pain in ADPKD is often unable to be determined compared with other general pain [10].

The Standardized Outcomes in Nephrology-PKD (SONG-PKD) initiative identified pain as one of the four core outcomes in PKD [11], defined as outcomes of critical importance to all key stakeholders, including patients/caregivers, health professionals, policy makers/funders from recently completed consensus workshop. Pain is the only patient-reported outcome (PROM) in the core outcome set, which was highly prioritised due to its significant and adverse impact on daily and social activities [12]. Despite being identified as a critically important outcome [13], pain is often under recognized and poorly managed [13]. Although there are strategies available to manage pain, including non-pharmacologic treatments (management of diet and lifestyle), analgesics and surgery [14], people with ADPKD still report pain and there is no a systematic approach for the clinical assessment of pain in this setting [3].

Despite its critical importance and being highly prioritized by all stakeholders, pain was reported in only 16 (24%) of randomized trials involving people with ADPKD according to a recent systematic review [15]. Pain has been identified as a core outcome in ADPKD, which means it is to be measured and reported in all trials involving people with ADPKD, using a consistent, validated outcome measurement tool. The aim of this study was to identify the content, general characteristics, and validity of measures used to assess pain in people with ADPKD, to select a robust and feasible outcome measure to use in all clinical trials in this setting. The identification of a suitable measure to capture crucial aspects of pain will ensure an accurate assessment and better understanding of factors associated with pain, and identify interventions targeting to manage pain in people with ADPKD.

## Materials and methods

### Selection criteria

We searched for all study designs (interventional and non-interventional studies) that involved patients aged at least 18 years with ADPKD and included a patient-reported outcome measure (questionnaire) that assessed any type of pain. Global measures (e.g. a composite measure for health-related quality of life or health status that assessed multiple domains including pain) were included if they reported a pain-specific item, even if they were not designed for ADPKD population. However, only items related to pain were assessed. Studies published in peer-reviewed journals without language restrictions were included. Abstract-only citations were included only if we were able to extract sufficient information about the measure (characteristics and content) used to assess pain.

### Study sources and measures

We conducted searches in MEDLINE, Embase, PsycINFO and the CKT register from database inception to February 2020. Google Scholar and reference lists of relevant studies and reviews were also searched. The search strategies are provided in S1 Table. Three authors (PN, EH, AT) independently screened all abstracts and excluded those not meeting the inclusion criteria, then assessed remaining full-text articles for eligibility. Any uncertainties and disagreements about the inclusion of articles were discussed until we reached consensus.

### Data extraction and analysis

We extracted the following characteristics from each study included: publication year, country, study design, sample size, type of intervention (if applicable), measure used to assess pain and study duration. To summarize characteristics for the measures identified, PN and EH referred to the source study and key references to extract the following information: response format scale, number of items, recall period, cost of license to use the measure, completion time, language and number of studies that used the measure. PN performed a distinct search for validation studies for each measure and extracted psychometric data in people with ADPKD, using the Consensus-based Standards of health Measurement Measures-Core outcome measures in Effectiveness Trials (COSMIN-COMET) [16]. The data were independently cross-checked by authors EH and AB.

### Dimensions of pain

To determine the dimensions assessed in the measures of pain, PN and EH initially extracted all items (questions) on pain, including items from the pain subscale of global measures. The range of items were sorted into dimensions inductively derived. The dimensions identified were the site of pain, measurement (e.g. frequency, intensity), type of pain, and impact of pain. The frequency of each dimension was recorded.

### Assessment of psychometric properties

As recommended by COSMIN-COMET [16] guidelines, we examined the available evidence for validity and reliability of the identified measures by examining psychometric properties: content validity, structural validity, criterion validity, reliability including test-retest and internal consistency, measurement error, cross-cultural validity and responsiveness.

## Results

### Characteristics of studies

**Fig 1:**
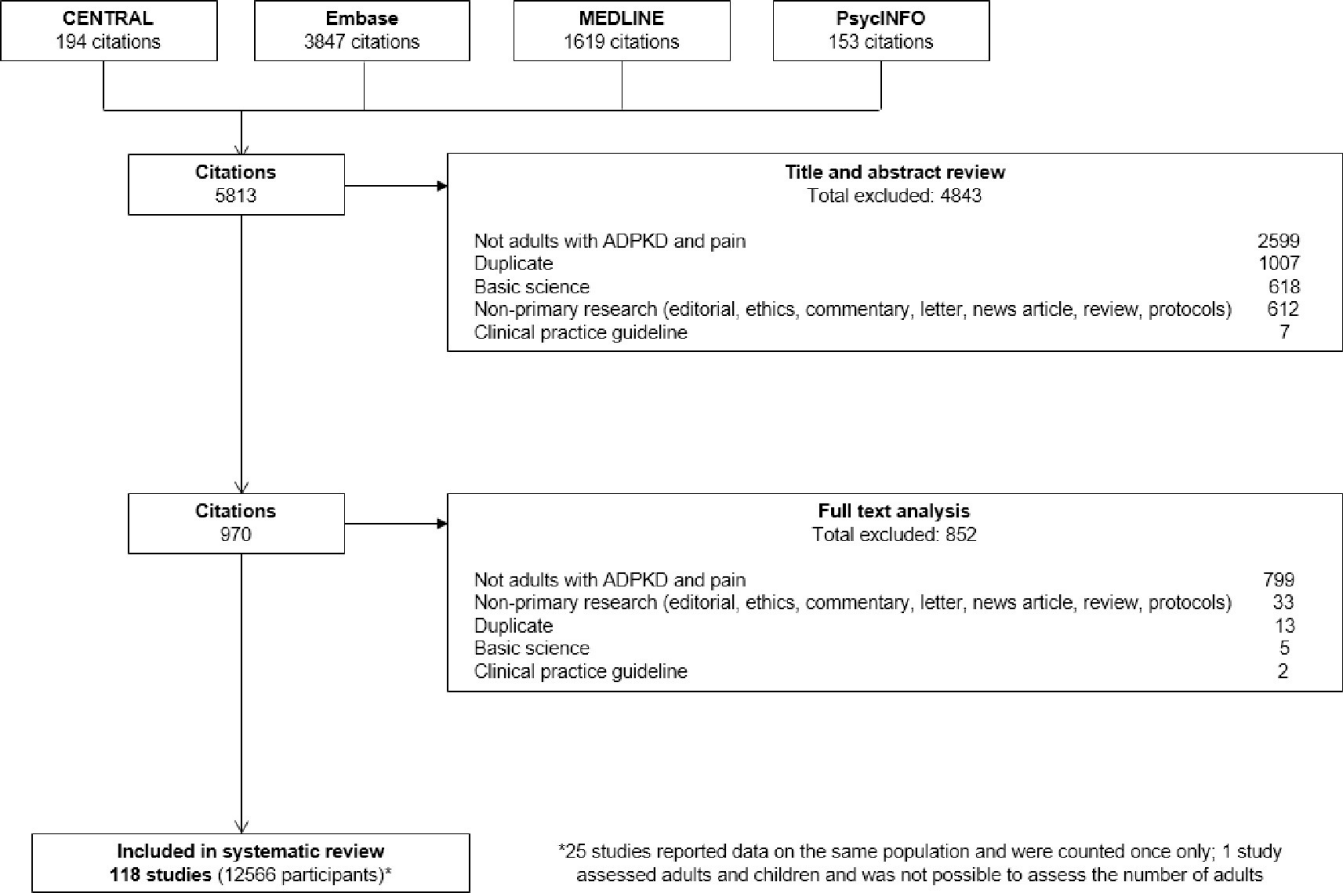
PRISMA flowchart.

We identified 4806 potential relevant citations after removing the duplicates. We included 118 studies involving a total of 12,566 participants with ADPKD across 35 countries. Of the included studies, 36 (30%) were interventional studies and 82 (70%) were non-interventional studies. The search results are shown in Fig 1. The study characteristics and measures used to assess pain are provided in S2 and S3 Tables. The PRISMA Checklist is provided in S6 Table.

### Characteristics of the measures

**Fig 2:**
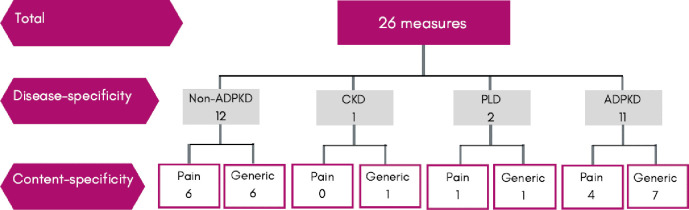
Type of measures used to assess pain in patients with ADPKD.

Across the 118 studies, there were 26 different patient-reported outcome measures used to assess pain. Of these, 16 (62%) were established measures and 10 (38%) measures were designed by the authors for use in their study only, without validation. Twelve (46%) measures were developed for a non-ADPKD population, one (4%) for all stages of chronic kidney disease, two (8%) for polycystic liver disease and 11 (42%) were developed for people with ADPKD (Fig 2). Eleven (42%) measures were developed specifically to assess general pain and 15 (58%) assessed broader outcomes such as quality of life and symptoms, in which pain was a subscale. Regarding measures that were not specifically designed to assess pain, number of items ranged from one (n = 4 measures [17–20]), two (n = 3 measures [21–23]), three (n = 5 measures [24–28]), four (n = 1 measure [29]), five (n = 1 measure [30]), and 10 items (n = 1 measure [31]).

Type of measures used to assess pain in patients with ADPKD. Abbreviations and definitions: non-ADPKD, measures developed for patients without autosomal dominant polycystic kidney disease; CKD, measures developed for patients with chronic kidney disease; PLD, measures developed for patients with polycystic liver disease; ADPKD, measures developed for patients with autosomal dominant polycystic kidney disease.

The 36-Item Short Form Health Survey (SF-36) was the most frequently used measure reported in 16 (33%) studies, followed by Visual Analog Scale (VAS) in 11 (22%) studies, EuroQol-5 Dimension Questionnaire (EQ-5D) in seven (14%) studies and Gastrointestinal questionnaire (GI-Q) in six (12%) studies. The time taken for completion of each measure ranged from two minutes to 30 minutes.

The number of items in the questionnaires varied from nine (Short-form Brief Pain Inventory (BPI-SF)) to 80 (Kidney Disease Quality of life—Short Form (KDQOL-SF)). The recall period ranged from the day of assessment to 10 years. Most of the measures (23, 88%) were free of charge for non-commercial use, some of which required study registration. Characteristics of measures and frequency of use are provided in Table 1.

**Table 1:** Characteristics of the measures.

Measure	Response format scale	N of items	Recall period	Cost of license	Completion time (min)	Pain specific	CKD specific	PLD specific	ADPKD specific	Language	Frequency of use (number of studies)
**Established measures**											
SF-36 [22]	Y/N, 3-/5-/6- point Likert	36	Various	Annual license fee ^a^	10–15 min	No	No	No	No	English, Italian, German, Japanese	16 (33%)
VAS ^b^	0–10 numerical scale	-	Before/after treatment (up to 120 months)	Contact author	-	Yes	No	No	No	English	11 (22%)
EQ-5D [20]	VAS	16	Various	Licensing fee based on quote	<5 min	No	No	No	No	English, Danish, Dutch, Swedish	7 (14%)
GI-Q [27]	7-point Likert	11	Various	Contact author	~2–3 min	No	No	No	No	-	6 (12%)
KDQOL-SF [29]	Y/N, 3-/4-/5-/6 point Likert	80	Various	Free	~30 min	No	Yes	No	No	Italian	2 (4%)
ADPKD-IS [24]	5-point Likert	18	Various	Contact author	<5 min	No	No	No	Yes	English	2 (4%)
PLD-Q [31]	Numerical scale	13	Before/after treatment	Contact author	<5 min	No	No	Yes	No	Dutch, English	2 (4%)
EORTC QLQ-C30 [23]	4-point Likert	30	Before/after treatment	License fee for non-academic users depends on number of patients	~6 min	No	No	No	No	Dutch, English	2 (4%)
BPI-SF [32]*	Y/N; 0–10 numerical scale	9	Before/after treatment	Contact author	~2 min	Yes	No	No	No	English	1 (2%)
MPQ-DV [33]	VAS	17	Before/after treatment (up to 12 months)	Contact author	2–5 min	Yes	No	No	No	Dutch,	1 (2%)
SF-MPQ-2 [34]*	VAS	22	Before/after treatment	Contact author	2–5 min	Yes	No	No	No	English	1 (2%)
SF-12 [18]	Y/N; 3-,5-,6-point Likert	12	Current	License fee upon request	~2–3 min	No	No	No	No	English, Danish, Swedish	1 (2%)
GSRS (Revised) [28]	2-,4-point Likert	11	Current	Contact author	~2–3 min	No	No	No	Yes	Korean	1 (2%)
HAM-D [19]	3-,5-poin Likert	21	Before/after treatment (up to 6 months)	Contact author	15–20 min	No	No	No	No	-	1 (2%)
Wisconsin BPS (Revised) [6]	6-point Likert	-	Current	Contact author	-	Yes	No	No	Yes	English	1 (2%)
GI-Q (Revised for PLD) [35]	7-point Likert	11	Before/after treatment (up to 30 months)	Contact author	~2–3 min	Yes	No	Yes	No	-	1 (2%)
**Author-developed measures**											
D’Agnolo 2016 [36]^#^^!^^c^	1–10 numerical scale	-	Current	Contact author	-	Yes	No	No	Yes	-	2 (4%)
D’Agnolo 2017 [37]^!^^c^											
(ADPKD-related pain)											
Torres 2012 [7]^!^^d^	0–10 numerical scale	-	Before/after treatment (up to 36 months)	Contact author	-	Yes	No	No	Yes	Multiple languages	2 (4%)
Torres 2011 [38]^!^^d^											
(Kidney pain score)											
Suwabe 2013 [21]	4-, 5-point Likert	12	Current	Contact author	~2–3 min	No	No	No	Yes	Japanese	1 (2%)
Suwabe 2017 [25]	4-, 5-point Likert	15	Before/after treatment (up to 12 months)	Contact author	<5 min	No	No	No	Yes	Japanese	1 (2%)
Haseebuddin 2012 [39]	-	-	After treatment	Contact author	-	Yes	No	No	Yes	English	1 (2%)
Iliuta 2019 [26]	5-point Likert	-	Before/after treatment	Contact author	-	No	No	No	Yes	English	1 (2%)
Sakuhara 2015 [17]	0–10 numerical scale	-	Before/after treatment (up to 24 months)	Contact author	-	No	No	No	Yes	-	1 (2%)
Taylor 2005 [30]	Y/N	-	Current	Contact author	-	No	No	No	Yes	English	1 (2%)
Abraham 2015 [40]	Numerical scale	-	First 48 hours	Contact author	-	Yes	No	No	No	-	1 (2%)
Walsh 2012 [41]	0–10 numerical scale	-	-	Contact author	-	Yes	No	No	No	English	1 (2%)

(-) Not stated, unclear, or unable to ascertain

* The name of questionnaire was extracted from the protocol, since it was not clearly stated in the primary publication (only “Pain questionnaire” was reported)

# Abstract

! More than one studied referred to the same publication; Y/N: Questions requiring yes/no answers. Note: The language is referred to the language that was used in the studies rather than the available languages for the measures.

Abbreviations: KDQOL-SF: Kidney Disease Quality of life—Short Form; GI-Q: Gastrointestinal questionnaire; VAS: Visual Analog scale; SF-36: Medical Outcomes Study Form, 36 items health survey; ADPKD-IS: ADPKD Impact Scale; PLD-Q: Polycystic Liver Disease questionnaire; EQ-5D: European Quality of Life-5 Dimension Questionnaire that may include a VAS scale for pain; EORTC QLQ-C30: European Organization for Research and Treatment of Cancer quality of life questionnaire core-30; BPI-SF: Short-form Brief Pain Inventory; SF-MPQ: Short Form of the McGill Pain Questionnaire; MPQ-DV: McGill Pain Questionnaire (Dutch version); SF-12: Health Survey Short Form 12; GSRS: Gastrointestinal Symptom Rating Scale; HAM-D: The Hamilton Depression Rating Scale; Wisconsin BPS: Modified version of Wisconsin Brief Pain Survey.

^a^ Upon registration for version 2, users can obtain a quote for the license fee that applies to their project; version 1 can be obtained for free

^b^ VAS scale was clearly reported to assess pain in all studies except Qian 2015 [42]. VAS item with open-ended response questions vary

^c^ This author-developed instrument was reported in two studies as ADPKD-related pain

^d^ This author-developed instrument was reported in two studies as Kidney pain score.

### Dimensions of pain

Pain-related items from the measures were classified into four content dimensions [43], which capture the site of pain (e.g. abdomen, lower back, thorax, head and face, generalized body and non-specified), measurement (intensity, frequency, temporality and sensory), type (nociceptive and neuropathic pain) and impact (life participation, sleep and mental).

The measures assessed 10 sites dimensions: abdomen including general abdomen (8 [31%] measures), upper abdomen (4 [15%]), lower abdomen (2 [8%]), flank (7 [27%]); lower back (10 [39%]); thorax including chest (1 [4%]) and rib cage (2 [8%]); head and face (3 [12%]); generalized body (8 [31%]); and non-specified pain (9 [35%]). The measures assessed four measurement dimensions: intensity (23 [88%]), frequency (3 [12%]), temporality (2 [8%]), and sensory (21 [81%]); and two dimensions for type: nociceptive including visceral (15 [58%]) and somatic (5 [20%]); and neuropathic (2 [8%]).

The measures assessed 12 impact dimensions that were reported only in the established measures: life participation including daily activity (4 [25%]), social activity (3 [19%]), work (5 [31%]), walking ability (2 [13%]), physical function (2 [13%]), and strenuous physical activity (1 [6%]), and one dimension for sleep (3 [19%]). The measures assessed mental impact including mood (2 [13%]), bother (2 [13%]), anxiety (1 [6%]), affective (2 [13%]), and enjoyment of life (2 [13%]). None of the author-developed measures assessed impact dimensions. The definitions of each dimension are given in Tables 2–4. Dimensions of pain assessed by measures are provided in S5 Table.

**Table 2:** Dimensions of pain assessed by measures: Site of pain.

Measure	Site									
	Abdomen				Lower back*	Thorax		Head and Face	Generalized body	Non-specified
	General	Upper abdomen	Lower abdomen	Flank		Chest	Rib cage (behind/below)^^^			
**Established measures**										
**SF-36 [22]**^**c**^									**●**	
**VAS** ^**d**^^,^^**f**^^,^^**g**^^,^^**h**^^,^^**i**^^,^^**j**^^,^^**k**^^,^^**s**^	**●**			**●**	**●**					**●**
**EQ-5D [20]**^**d**^										**●**
**GI-Q [27]**^t^		**●**	**●**						**●**	
**KDQOL-SF [29]**^**b**^^,^^**c**^						**●**			**●**	
**ADPKD-IS [24]**^**o**^										**●**
**PLD-Q [31]**^**n**^	**●**			**●**	**●**		**●**			
**EORTC QLQ-C30 [23]**^**d**^										**●**
**BPI-SF [32]**^**d**^								**●**		**●**
**MPQ-DV [33]**^**r**^										**●**
**SF-MPQ-2 [34]**^**o**^										**●**
**SF-12 [18]**										**●**
**GSRS (Revised) [28]**^**l**^^,^^**p**^		**●**		**●**	**●**				**●**	
**HAM-D [19]**^**b**^					**●**			**●**	**●**	
**Wisconsin BPS (Revised) [6]**	**●**				**●**					
**GI-Q (Revised for PLD) [35]**		**●**	**●**							
**Author-developed measures**										
**D’Agnolo 2016 [36]**^**#**^^**!**^	**●**	**●**		**●**	**●**		**●**		**●**	
**D’Agnolo 2017 [37]**^**!**^^**m**^^,^^**p**^										
**Torres 2012 [7]**^**!**^	**●**			**●**	**●**					
**Torres 2011 [38]**^**!**^^**a**^										
**Suwabe 2013 [21]**^**p**^									**●**	
**Suwabe 2017 [25]**^**q**^									**●**	
**Haseebuddin 2012 [39]**									**●**	
**Iliuta 2019 [26]**	**●**			**●**	**●**					
**Sakuhara 2015 [17]**	**●**				**●**					
**Taylor 2005 [30]**^**e**^	**●**			**●**	**●**			**●**		**●**
**Abraham 2015 [40]**									**●**	
**Walsh 2012 [41]**									**●**	

* Lower back pain was attributed to kidney pain

^ Pain behind or below the rib cage was attributed to liver pain

# Abstract

! More than one studied referred to the same publication

**●** Data were available for the selected field.

^a^ Measure reported kidney pain without other specific description. According to the definition reported in other studies, kidney pain was described as flank, back and abdominal pain

^b^ Generalized pain included muscle aches

^c^ Generalized pain included bodily pain

^d^ Qian 2015 [42] non-specified if VAS was a “pain specific instrument”, and no any additional information were reported about pain

^e^ Other pain was assessed as non-specified pain

^f^ Chrispijn 2013 [27] used VAS scale used to assess pain to record abdominal pain

^g^ Kucuk 2016 [44], Dunn 2000 [45], Lee 2003 [46], Lee 2004 [47] reported that patients had abdominal pain and flank pain. Although no further information were reported about VAS scale used to assess pain, we can assume that authors used this instrument to assess abdominal and flank pain

^h^ Dunn 2001 [48] reported that patients had abdominal pain. Although no further information were reported about VAS scale used to assess pain, we can assume that authors used this instrument to assess abdominal pain

^i^ Lipke 2007 [49], Yu 2018 [50] reported that patients had kidney pain. Although no further information were reported about VAS scale used to assess pain, we can assume that authors used this instrument to assess kidney pain

^j^ Rehman 2001 [51] reported that patients had flank pain with or without back pain

^k^ Sulikowski 2006 [52] reported that patients had back and abdominal pain. Although no further information were reported about VAS scale used to assess pain, we can assume that authors used this instrument to assess abdominal and back pain

^l^ Upper abdomen pain included epigastric soreness and right upper abdomen quadrant pain

^m^ Upper abdomen pain was reported as right upper abdomen pain, behind or below the rib cage, and was attributed to liver pain

^n^ Rib cage pain was reported as pain or pressure in the rib cage

^o^ Pain location was not reported

^p^ Generalized pain included heartburn

^q^ Generalized pain included bodily pain and heartburn

^r^ Pain location was not reported

^s^ VAS item with open-ended response questions vary

^t^ Generalized pain included heartburn and epigastric pain.

**Table 3:** Dimensions of pain assessed by measures: Measurement and type of pain.

Measure	Measurement				Type		
	Intensity	Frequency	Temporality*	Sensory^§^	Nociceptive		Neuropathic
					Visceral^^^	Somatic^$^	
**Established measures**							
**SF-36 [22]**^**s**^	**●**			**●**			
**VAS** ^**s**^^,^^**ç**^^,^ ^**£**^	**●**		**●**	**●**			
**EQ-5D [20]**^**s**^	**●**			**●**			
**GI-Q [27]**^**a**^^,^^**q**^	**●**			**●**	**●**		
**KDQOL-SF** ^**[**^^**29**^^**]**^^**b**^^,^^**s**^^,^^**t**^	**●**			**●**	**●**	**●**	
**ADPKD-IS [24]**	**●**						
**PLD-Q [31]**^**c**^^,^^**r**^		**●**		**●**	**●**	**●**	
**EORTC QLQ-C30 [23]**^**s**^	**●**			**●**			
**BPI-SF [32]**^**d**^^,^^**u**^	**●**			**●**		**●**	
**MPQ-DV [33]**^¥^	**●**			**●**			
**SF-MPQ-2 [34]**^**e**^			**●**	**●**			**●**
**SF-12 [18]**	**●**						
**GSRS (Revised) [28]**^**f**^^,^^**r**^	**●**	**●**		**●**	**●**		
**HAM-D [19]**^**g**^^,^^**r**^^,^^**t**^	**●**			**●**	**●**	**●**	
**Wisconsin BPS (Revised) [6]**^**h**^^,^^**r**^^,^^**z**^	**●**	**●**		**●**	**●**		**●**
**GI-Q (Revised for PLD) [35]**^**i**^^,^^**q**^	**●**			**●**	**●**		
**Author-developed measures**							
**D’Agnolo 2016 [36]**^**#**^^**!**^	**●**				**●**		
**D’Agnolo 2017 [37]**^**!**^^**j**^							
**Torres 2012 [7]**^**!**^	**●**				**●**		
**Torres 2011 [38]**^**!**^^**k**^							
**Suwabe 2013 [21]**^**l**^^,^^**q**^^,^^**r**^	**●**			**●**	**●**		
**Suwabe 2017 [25]**^**m**^^,^^**q**^^,^^**r**^^,^^**s**^	**●**			**●**	**●**		
**Haseebuddin 2012 [39]**^**s**^^,^^**v**^	**●**			**●**			
**Iliuta 2019 [26]**^**n**^^,^^**r**^	**●**			**●**	**●**		
**Sakuhara 2015 [17]**^**o**^^,^^**r**^	**●**			**●**	**●**		
**Taylor 2005 [30]**^**p**^					**●**	**●**	
**Abraham 2015 [40]**^**s**^	**●**			**●**			
**Walsh 2012 [41]**^**s**^	**●**			**●**			

* Temporality included continuous, recurring, irregular, intermittent pain

§ Sensory included the sensation of pain as burning, sharp, intense, aching

^Visceral pain included injury/damage to internal organs (liver, kidney), abdomen, chest, epigastric soreness, heartburn

^$^ Somatic pain included injury/damage to muscle, rib cage, headaches, sprains, cramps

# Abstract

! More than one studied referred to the same publication

^£^ VAS item with open-ended response questions vary

**●** Data were available for the selected field.

^a^ Visceral pain was defined as lower and upper abdominal pain and heartburn

^b^ Visceral pain was defined as chest pain. Somatic pain was defined as cramps

^c^ Visceral pain was defined as back, flank and abdominal pain. Somatic pain was defined as pain or pressure in rib cage

^d^ Somatic pain was defined as headaches, sprains

^e^ Sensory descriptors of pain included:1) continuous pain descriptors (throbbing, cramping, gnawing, aching, heavy and tender pain); 2) intermittent pain descriptors (shooting, stabbing, sharp, splitting, electric-shock and piercing pain); 3) predominantly neuropathic pain descriptors (hot-burning and cold-freezing pain, pain caused by light touch, itching, tingling or pins and needles, numbness

^f^ Visceral pain was defined as right upper quadrant, back, flank pain and epigastric soreness

^g^ Visceral pain was defined as back pain. Somatic pain was defined as muscle pain, headaches and cramps

^h^ Visceral pain was defined as back and abdominal pain

^i^ Visceral pain was defined as lower and upper abdominal pain and heartburn

^j^ Visceral pain was defined as liver (defined as right upper quadrant pain, behind or below the rib cage) and kidney pain (defined as back, flank and abdominal pain)

^k^ Visceral pain was defined as kidney pain

^l^ Visceral pain was defined as heartburn

^m^ Visceral pain was defined as heartburn

^n^ Visceral pain was defined as back, flank and abdominal pain

^o^ Visceral pain was defined as back and abdominal pain

^p^ Visceral pain was defined as back, flank and abdominal pain. Somatic pain was defined headaches

^q^ Heartburn was assessed as sensory pain

^r^ Abdominal distension/fullness/ heavy feelings in abdomen were assessed as sensory pain

^s^ Bodily/generalized pain was assessed as sensory pain

^t^ Cramps and was assessed as sensory pain

^u^ Sprains was assessed as sensory pain

^v^ Pain intensity was reported as degree of subjective pain relief

^z^ Neuropathic pain was reported as radicular pain

^ç^ Rehman 2001 [51] reported intermittent pain

^¥^ Sensory descriptors of pain included temporal, spatial, punctate pressure, incisive pressure, constrictive pressure, traction pressure, thermal, dullness, stiffness, continuity.

**Table 4:** Dimensions of pain assessed by measures: Impact of pain*.

Measure	Impact											
	Life participation						Sleep	Mental				
	Daily activity	Social activity	Work^$^	Walking ability	Physical function	Strenuous physical activity		Mood	Bother	Anxiety	Affective	Enjoyment of life
**Established measures**												
**SF-36 [22]**			**●**									
**VAS** ^**£**^												
**EQ-5D [20]**												
**GI-Q [27]**												
**KDQOL-SF [29]**			**●**									
**ADPKD-IS [24]**^**a**^	**●**				**●**		**●**		**●**			
**PLD-Q [31]**									**●**			
**EORTC QLQ-C30 [23]**	**●**											
**BPI-SF [32]**^**b**^	**●**	**●**	**●**	**●**			**●**	**●**				**●**
**MPQ-DV [33]**^**d**^											**●**	
**SF-MPQ-2 [34]**^**c**^											**●**	
**SF-12 [18]**			**●**									
**GSRS (Revised) [28]**		**●**										
**HAM-D [19]**										**●**		
**Wisconsin BPS (Revised) [6]**^**b**^	**●**	**●**	**●**	**●**	**●**	**●**	**●**	**●**				**●**
**GI-Q (Revised for PLD) [35]**												

* No author-developed measures reported impact as a dimension of pain

^$^ Work included both work outside the home and housework

^£^ VAS item with open-ended response questions vary

**●** Data were

available for the selected field. Note: Physical function included normal or mild exercises.

^a^ Daily activity included the patient’s need to modify lifestyle

^b^ Social activity included relations with others and hobbies

^c^ Affective descriptors of pain included tiring-exhausting, sickening, fearful and punishing-cruel

^d^ Affective descriptors of pain included tension, autonomic, fear, punishment.

### Psychometric properties

The validity and reliability for each measure in people with ADPKD is shown in S4 Table. Of the 16 measures, only two (13%) were validated in the ADPKD population. A summary of the psychometric data for each of these measures is shown in Table 5. The validation data for the measures were variable and only one measure, the ADPKD Impact Scale (ADPKD-IS) [24], provided information across all psychometric domains. The Polycystic Liver Disease questionnaire (PLD-Q) [31] was also evaluated for psychometric properties in people with ADPKD.

**Table 5:** Development/validation data on psychometric properties.

Measure/Psychometric proprerty	Validity						Reliability	
	Content	Construct			Criterion		Test-retest	Internal consistency
			Convergent	Discrimination	Predictive	Concurrent		
**ADPKD-IS [24]**	**●**	**●**		**●**		**●**	**●**	**●**
**PLD-Q [31]**	**●**	**●**		**●**		**●**	**●**	**●**

Note: Validation studies were excluded if they were not available in full, were for a translation of the original measure or were not written in English

**●** Data were available for the selected field.

The ADPKD-IS was a 18 items measure created specifically for the ADPKD population, and the development of the measure included quantitative and qualitative studies, with the involvement of clinicians with expertise in ADPKD and people with ADPKD [24]. Confirmatory Factor Analysis supported a three-factor structure rather than the hypothesised two-factor structure, with the addition of four domain-independent items that were retained in the measure. Item discrimination for all items was adequate and each item was adequately correlated to its respective domain, supporting construct validity. Discriminant validity was supported through correlations between changes in the physical domain score and changes in pain intensity measured by pain ratings in the BPI-SF. As predicted, higher scores on all domains were associated with more advanced disease stage. Correlations between ADPKD-IS domains and summary scores on the Health Survey Short Form 12 (SF-12) indicated sufficient concurrent validity. The ADPKD-IS has high test-retest reliability and high internal consistency, both overall and within each domain [24].

The PLD-Q was not created for ADPKD patients without polycystic liver disease, however people with ADPKD were included in the validation study. The PLD-Q was designed based on qualitative and quantitative studies, including two validation studies. Factor analysis supported a unidimensional structure of the measure. As expected, ADPKD patients scored higher for pain than healthy controls but lower than patients with PLD, supporting discriminant validity [31]. Concurrent validity was supported by the high correlation between total PLD-Q score and the European Organization for Research and Treatment of Cancer quality of life questionnaire core-30 (EORTC QOL30) symptom burden score, as well as between the total PLD-Q and the VAS global health scale. Test-retest reliability and internal consistency were both high.

## Discussion

The measures of pain differed in content, length, response format scale, number of items, recall period, cost of licence, completion time and available psychometric proprieties. Our analysis showed that only 119 studies in people with ADPKD measured pain, and of these 36 (30%) were randomized trials. Across these studies, 26 different measures were used, which demonstrates inconsistencies in how pain in ADPKD is assessed. Only 11 (42%) were developed for an ADPKD population, of which most (eight measures) were non-validated and developed *de novo* for the author’s study. More than half of the measures (58%) were not designed specifically for pain. Over one-third of measures (35%) had at least 15 items and four (15%) measures had an estimated completion time of more than five minutes. The sites of pain included lower back and non-specified region/s, and the measurement properties including intensity and types were reported in at least one-third of the measures. The impact of pain on life participation and mental wellbeing were assessed in 12 (75%) measures, and all in established measures only.

Evidence for psychometric validation of the measure for pain in the ADPKD was available for only two measures. Two measures [24, 31], including one that was designed for PLD, assessed psychometric robustness in ADPKD based on the COSMIN-COMET properties. One measure reported data on all psychometric domains in ADPKD. Both measures had more than 10 items and costs of licence were not reported, and they might not be feasible as a core outcome measure or applicable in low income countries. The suitability of other measures to evaluate pain in ADPKD remains unclear as content validity and internal consistency may not be transferable across different patient populations because of range of clinical conditions and treatments. Pain is infrequently evaluated as a separate construct in ADPKD, and often it is incorporated as an item of health-related quality of life. Our findings suggest that pain has been not adequately assessed and a standardized and psychometrically valid measure that addresses key dimensions of pain in ADPKD is needed. However, it is still unclear if a new ADPKD-specific measure may be more appropriate compared to the current measures.

The current measures show a very broad range of dimensions in terms of the site of pain, measurement properties, type and impact. Pain complaints are complex and some patients with ADPKD develop a so called chronic pain syndrome, including chronic pelvic pain syndrome or irritable bowel syndrome, that can exacerbate pain. Many of these measures do not reflect the dimensions of pain important to people with ADPKD, are burdensome to administer and complete, and lack evidence to support the psychometric properties. It remains unclear whether some of these measures were able to discriminate between kidney specific and not kidney specific pain, and central sensitization and mental status have not been adequatelly investigated. The pain subscale in the SF-36 questionnaire, which was the most commonly used measure, included only two questions and it is unclear if they assess the dimensions of critical importance to people with ADKPD, and the specific subscale has not been validated. Pain should be collected in “real-time” using tools such as mobile apps, especially because people with ADPKD have both a chronic level of pain and experience acute pain episodes which have to be adequately distinguished.

This review yields a comprehensive assessment of measures that have been used to evaluate pain in people with ADPKD. However, there are some potential limitations. We did not include study protocols and unpublished studies, so some measures of pain used in people with ADPKD may not have been captured and other measures could be potentially adequate to use in ADPKD setting (e.g. the Central Sensitization Inventory [53]). We searched for primary validation studies and did not conduct systematic search for the validation studies for each measure. We recognize that it would not be possible to find all relevant studies that reported validation data for all the measures, and there are only limited data on the psychometric properties because of the limited number of measures validated in ADPKD. We acknowledge that people with ADPKD experience frequently adaptation to recurrent acute and chronic pain and, despite the severity of symptoms, the real impact of pain could be underestimated in this setting.

To improve consistency in reporting patient-important outcomes such as pain, core outcome measures for pain have been established in other non-CKD patient populations. In rheumatology, as part of the Outcome Measures in Rheumatology (OMERACT) [54] initiative, focus groups and interviews were conducted with patients and clinical researchers to establish patient-relevant outcome domains and to identify a measure that captured all critical aspects to assess pain in patients with hip or knee osteoarthritis [55]. However, there were no optimal existing measures in osteoarthritis and a new measure was developed addressing intensity, frequency, impact on quality of life and sleep, and the extent of worry and frustration on pain recurrence, which were of importance to patients [56]. Several PROMIS pain measures have been established and demonstrated to be psychometrically robust in other chronic conditions, such as cancer [57]. Although these measures have not been validated in ADPKD and did not provide further dimensions, the PROMIS item bank for pain can be considered as a reference for the list of possible questions to evaluate in each dimension, considering intensity and life participation as essential dimensions also in our setting. The impact of pain in sexual intercourse and central sensitization have not been explored and may be included in the future research.

As part of the SONG-PKD initiative, pain was established as a core outcome based on a consensus among patients, caregivers and health professionals [12]. This systematic review is the first phase in identifying or developing a standardized, validated measure for pain, and provides detailed and comprehensive information about the domains to potentially address when measuring pain in people with ADPKD. Subsequent work will be based on the COMET-COSMIN framework [16] for establishing core patient-reported outcome measures and will involve an international consensus workshop with patients with ADPKD and health professionals, to discuss potential patient-reported outcome measures for pain to capture all of the relevant domains that are considered important to patients. The proposed measure will be piloted and validated in people with ADPKD, to assess if the measure is appropriate and reliable to evaluate pain in this setting. A standardized and validated measure to assess pain in people with ADPKD will improve consistency in the assessment and reporting of pain in research, and may lead to better pain management and patient outcomes.

## Supporting information

S1 Table Search strategies. (DOCX)

S2 Table Characteristics of interventional studies. (DOCX)

S3 Table Characteristics of the non-interventional studies. (DOCX)

S4 Table Psychometric properties of measures that have been used to assess pain in patients with ADPKD. (DOCX)

S5 Table Dimensions of pain assessed by measures used in patients with ADPKD. (DOCX)

S6 Table PRISMA checklist. (DOC)
