# Supplementary material for: Patient-reported outcome measures for pain in autosomal dominant polycystic kidney disease: A systematic review
Source: PLoS One. 2021 May 27;16(5):e0252479. doi: 10.1371/journal.pone.0252479 (PMC8158964; doi:10.1371/journal.pone.0252479)
Supplement: S1 Table — (DOCX) [file pone.0252479.s001.docx]

**S1 Table. Search strategies**

**MEDLINE 1946 to 14^th^ February 2020**

| 1 | Polycystic Kidney Diseases/ |
| --- | --- |
| 2 | Polycystic Kidney, Autosomal Dominant/ |
| 3 | polycystic kidney disease*.tw. |
| 4 | ADPKD.tw. |
| 5 | PKD.tw. |
| 6 | or/1-5 |
| 7 | randomized controlled trial.pt. |
| 8 | controlled clinical trial.pt. |
| 9 | pragmatic clinical trial.pt. |
| 10 | randomized.ab. |
| 11 | placebo.ab. |
| 12 | clinical trials as topic/ |
| 13 | randomly.ab. |
| 14 | (crossover or cross-over).tw. |
| 15 | Cross-over Studies/ |
| 16 | trial.ti. |
| 17 | or/7-16 |
| 18 | exp epidemiologic studies/ |
| 19 | Observational study/ |
| 20 | (cohort adj stud*).tw. |
| 21 | (follow up adj stud*).tw. |
| 22 | longitudinal.tw. |
| 23 | retrospective.tw. |
| 24 | cross sectional.tw. |
| 25 | (observational adj (study or studies)).tw. |
| 26 | or/18-25 |
| 27 | 17 or 26 |
| 28 | 6 and 27 |
| 29 | animals/ not (humans/ and animals/) |
| 30 | 28 not 29 |

**Embase 1974 to 14^th^ February 2020**

| 1 | Kidney Polycystic Disease/ |
| --- | --- |
| 2 | polycystic kidney disease*.tw. |
| 3 | ADPKD.tw. |
| 4 | PKD.tw. |
| 5 | or/1-4 |
| 6 | randomized controlled trial/ |
| 7 | crossover procedure/ |
| 8 | double-blind procedure/ |
| 9 | single-blind procedure/ |
| 10 | random$.tw. |
| 11 | factorial$.tw. |
| 12 | (crossover$ or cross-over$).tw. |
| 13 | placebo$.tw. |
| 14 | (double$ adj blind$).tw. |
| 15 | (double$ adj blind$).tw. |
| 16 | assign$.tw. |
| 17 | allocat$.tw. |
| 18 | or/5-17 |
| 19 | exp epidemiology/ |
| 20 | exp observational study/ |
| 21 | Case control.tw. |
| 22 | observational.tw. |
| 23 | (cohort adj stud*).tw. |
| 24 | (follow up adj stud*).tw. |
| 25 | longitudinal.tw. |
| 26 | retrospective.tw. |
| 27 | cross sectional.tw. |
| 28 | (observational adj (study or studies)).tw. |
| 29 | or/19-28 |
| 30 | 18 or 9 |
| 31 | 18 or 29 |
| 32 | 5 and 31 |
| 33 | or/20-28 |
| 34 | 18 or 33 |
| 35 | 5 and 34 |
| 36 | 5 and 18 |
| 37 | or/6-17 |
| 38 | 37 or 29 |
| 39 | 38 and 5 |

**PsycINFO** 1806 to 14^th^ February 2020

| 1 | polycystic kidney disease$.tw. |
| --- | --- |
| 2 | ADPKD.tw. |
| 3 | PKD.tw. |
| 4 | or/1-3 |
