# Supplementary material for: Patient-reported outcome measures for pain in autosomal dominant polycystic kidney disease: A systematic review
Source: PLoS One. 2021 May 27;16(5):e0252479. doi: 10.1371/journal.pone.0252479 (PMC8158964; doi:10.1371/journal.pone.0252479)
Supplement: S2 Table — (DOCX) [file pone.0252479.s002.docx]

**S2 Table. Characteristics of interventional studies**

| **Study ID** | **Publication year** | **Country** | **Study design** | **Sample size** | **Type of intervention**  **(if applicable)** | **Measure used to assess pain*** | **Study duration (months)^¥^** |
| --- | --- | --- | --- | --- | --- | --- | --- |
| **Randomized study** | | | | | | | |
| **Braun 2012[1] ^a^** | 2012 | Switzerland | Open label randomized controlled phase II trial (post hoc analysis) | 39 | Sirolimus (mTOR inhibitor) vs standard care | - | - |
| **Caroli 2013[2] ^b^**  **(ALADIN study)** | 2013 | Italy | Multicenter, randomized, single-blind, placebo-controlled, parallel-group trial | 79 | Octreotide (somatostatin analog) vs placebo | - | 36 |
| **Casteleijn 2017[3] ^c!^**  **(TEMPO 3:4 study)** | 2017 | Argentina, France, Australia, Italy, Belgium, Canada, Denmark, Japan, Germany, The Netherlands, Poland, USA, Romania, Russia, United Kingdom | Prospective, blinded, randomized, placebo-controlled trial (post hoc analysis) | 1445 | Tolvaptan (vasopressin V2 receptor antagonist) vs placebo | - | 36 |
| **Chrispijn 2013[4] ^d^** | 2013 | The Netherlands | Randomized, single-center, open-label, parallel clinical trial | 15^ | Everolimus (mTOR inhibitors) + octreotide (somatostatin analog) vs octreotide | EQ-5D; GI-Q; VAS ^u^ | 11 |
| **Chrispijn 2012[5] ^d^** | 2012 | The Netherlands | Open-label, observational extension study of a 6-month, randomized, placebo-controlled trial | 25^ | Lanreotide (somatostatin analog) vs placebo | SF-36; GI-Q | 12 |
| **D’Agnolo 2016[6] ^e^** | 2016 | The Netherlands and Spain | International, multicenter, randomized controlled phase 2 trial | 16^ | Ursodeoxycholic acid vs no treatment | SF-36; EQ-5D; PLD-Q; GI-Q; EORTC QLQ-C30 | 8 |
| **Davis 2018[7]** | 2018 | USA | Single-center, prospective, open-label, parallel-group, randomized trial | 23 | Sirolimus (mTOR inhibitor) + tacrolimus + prednisone vs mycophenolate + tacrolimus + prednisone | - | 12 |
| **Devuyst 2017[8] ^c!^**  **(TEMPO 3:4 study)** | 2017 | Argentina, France, Australia, Italy, Belgium, Canada, Denmark, Japan, Germany, The Netherlands, Poland, USA, Romania, Russia, United Kingdom | Randomized placebo controlled trial (post hoc analysis) | 1037 | Tolvaptan (vasopressin V2 receptor antagonist) vs placebo | - | 36 |
| **El-Damanawi 2019[9]**  **(DRINK study)** | 2019 | United Kingdom | Prospective single-center, open-label, randomized controlled Phase 2 trial | 42 | High water intake vs ad libitum intake | BPI-SF**; SF-MPQ-2**; EQ-5D | 2 |
| **EUDRA-CT 2010[10]**  **(TEMPO 4/4 study)** | 2010 | Argentina, France, Australia, Italy, Belgium, Canada, Germany, The Netherlands, Poland, USA, Romania, Russia, United Kingdom | Multi-center, open-label randomized controlled trial | 1083 | Tolvaptan (vasopressin V2 receptor antagonist) vs placebo | - | 24 |
| **Gansevoort 2019[11] ^c!^**  **(TEMPO 3:4 study)** | 2019 | Argentina, France, Australia, Italy, Belgium, Canada, Denmark, Japan, Germany, The Netherlands, Poland, USA, Romania, Russia, United Kingdom | Prospective, double-blinded, randomized placebo controlled trial (post hoc analysis) | 1280 | Tolvaptan (vasopressin V2 receptor antagonist) vs placebo | - | 36 |
| **Hogan 2012[12] ^g!^** | 2012 | USA | Randomized placebo-controlled clinical trial with open-label extension for one additional year when all patients took drug | 42 | Octreotide (somatostatin analog) vs placebo | SF-36 | 24 |
| **Hogan 2010[13] ^h!^** | 2010 | USA | Randomized double-blinded placebo-controlled clinical trial | 42 | Octreotide long-acting (somatostatin analog) vs placebo | SF-36 | 12 |
| **Irazabal 2016[14] ^c!^**  **(TEMPO 3:4 study)** | 2016 | Argentina, France, Australia, Italy, Belgium, Canada, Denmark, Japan, Germany, The Netherlands, Poland, USA, Romania, Russia, United Kingdom | Prospective, double-blinded, randomized placebo controlled trial (post hoc analysis) | 1445 | Tolvaptan (vasopressin V2 receptor antagonist) vs placebo | - | 36 |
| **Meijer 2018[15] ^l!^**  **(DIPAK-1 study)** | 2018 | The Netherlands | Multi-center, randomized, open-label, controlled clinical trial | 309 | Lanreotide (somatostatin analog) + standard care vs standard care | ADPKD-IS | 30 |
| **Muto 2015[16] ^m!^**  **(TEMPO 3:4 study)** | 2015 | Argentina, France, Australia, Italy, Belgium, Canada, Denmark, Japan, Germany, The Netherlands, Poland, USA, Romania, Russia, United Kingdom | Prospective, double-blinded, randomized placebo controlled trial (subgroup analysis) | 177 | Tolvaptan (vasopressin V2 receptor antagonist) vs placebo | - | 36 |
| **Muto 2017[17] ^c!^**  **(TEMPO 3:4 study Extension Japan trial)** | 2017 | Japan | Open-label, multicenter, phase III trial | 135 | Tolvaptan (vasopressin V2 receptor antagonist) vs placebo | - | 36 |
| **Perico 2019[18] ^n^**  **(ALADIN 2 study)** | 2019 | Italy | Randomized, double-blind, placebo-controlled, phase III multicenter trial | 100 | Octreotide (somatostatin analog) vs placebo | SF-36 | 36 |
| **Ruggenenti 2016[19] ^o^** | 2016 | Italy | Single-center, prospective, randomized, open label, blinded end point, parallel group trial | 41 | Sirolimus (mTOR inhibitor) + conventional therapy vs conventional therapy alone | - | 12 |
| **Schrier 2014[20] ^p!^**  **(HALT-PKD-A study)** | 2014 | USA | Double-blind, placebo-controlled trial | 558 | Lisinopril (angiotensin I–converting enzyme inhibitor) + telmisartan (angiotensin II–receptor blocker) vs Lisinopril + placebo | SF-36 | 96 |
| **Serra 2009[21] ^q!^**  **(SUISSE ADPKD study)** | 2009 | Switzerland | Ongoing single-center, prospective, open-label, randomized controlled clinical trial (interim analysis) | 50 | Sirolimus (mTOR inhibitor) vs standard care | - | 6 |
| **Serra 2010[22] ^q!^**  **(SUISSE ADPKD study)** | 2010 | Switzerland | Single-center, prospective, open-label, randomized controlled clinical trial | 100 | Sirolimus (mTOR inhibitor) vs standard care | - | 18 |
| **Tesar 2017[23] ^r^** | 2017 | Switzerland | Phase 2, multisite, randomized, double-blind, placebo-controlled study | 172 | Bosutinib (dual Src/Bcr-Abl tyrosine kinase inhibitor ) vs placebo | - | 50 |
| **Torres 2014[24] ^p!^**  **(HALT-PKD-B study)** | 2014 | USA | Randomized, double-blind, placebo-controlled trial | 486 | Lisinopril (angiotensin I–converting enzyme inhibitor) + telmisartan (angiotensin II–receptor blocker) vs Lisinopril + placebo | SF-36 | - |
| **Torres 2012[25] ^t!^**  **(TEMPO 3:4 study)** | 2012 | Argentina, France, Australia, Italy, Belgium, Canada, Denmark, Japan, Germany, The Netherlands, Poland, USA, Romania, Russia, United Kingdom | Phase 3, multicenter, double-blind, placebo-controlled, 3-year trial | 1445 | Tolvaptan (vasopressin V2 receptor antagonist) vs placebo | Torres 2012[25] | 36 |
| **Torres 2018[26] ^s!^**  **(TEMPO 4/4 study)** | 2018 | Argentina, France, Australia, Italy, Belgium, Canada, Germany, The Netherlands, Poland, USA, Romania, Russia, United Kingdom | Multi-center, Open-label, extension trial | 871 | Tolvaptan (vasopressin V2 receptor antagonist) vs placebo | - | 24 |
| **Torres 2017[27] ^s^** | 2017 | Argentina, France, Australia, Italy, Belgium, Canada, Germany, Israel, Hungary, The Netherlands, Norway, Puerto Rico, Poland, USA, South Africa, Spain, Sweden, Romania, Russia, United Kingdom, Czech, Denmark | Phase 3, randomized withdrawal, multicenter, placebo-controlled, double-blind trial | 1370 | Tolvaptan (vasopressin V2 receptor antagonist) vs placebo | - | 12 |
| **Torres 2016[28] ^c!^**  **(TEMPO 3:4 study)** | 2016 | Argentina, France, Australia, Italy, Belgium, Canada, Denmark, Japan, Germany, The Netherlands, Poland, USA, Romania, Russia, United Kingdom | Phase 3, multicenter, double-blind, placebo-controlled, 3-year trial (post hoc) | 1445 | Tolvaptan (vasopressin V2 receptor antagonist) vs placebo | - | 36 |
| **Torres 2011[29] ^c!^**  **(TEMPO 3:4 study)** | 2011 | Argentina, France, Australia, Italy, Belgium, Canada, Denmark, Japan, Germany, The Netherlands, Poland, USA, Romania, Russia, United Kingdom | Phase 3, multicenter, double-blind, placebo-controlled, 3-year trial (post hoc) | 1445 | Tolvaptan (vasopressin V2 receptor antagonist) vs placebo | Torres 2011[29] | 36 |
| **Van Aerts 2019[30] ^d!^**  **(DIPAK-1 study)** | 2019 | The Netherlands | Multi-center, randomized, open-label, controlled clinical trial | 175 | Lanreotide (somatostatin analog) vs standard care | GI-Q (Revised for PLD) | 30 |
| **Van Keimpema[31] 2009 ^d^** | 2009 | The Netherlands and Belgium | Randomized, double-blind, placebo-controlled trial | 32^ | Lanreotide (somatostatin analog) vs placebo | SF-36 | 6 |
| **Walz 2010[32] ^i^** | 2010 | Germany, Austria and France | Randomized, double-blind, placebo-controlled trial | 433 | Everolimus (mTOR inhibitor) vs placebo | - | 24 |
| **Non-randomized study** | | | | | | | |
| **Brioni 2019[33]** | 2019 | Italy | Prospective, longitudinal observational study (non-randomized) | 40 | Nursing-led education | KDQOL-SF | 9 |
| **Kucuk 2016[34] ^i^** | 2016 | Turkey | Non‑randomized retrospective pilot clinical study | 25 | Percutaneous aspiration and sclerotherapy treatment | VAS ^u^; SF-36 | 120 |
| **Lai 2020[35]** | 2020 | Italy | Controlled, longitudinal, prospective, interventional study | 59 | Alfa-lipoic acid vs control | HAM-D | 6 |
| **Makhlough 2017[36] ^k^** | 2017 | Iran | Single-arm phase I clinical trial | 6 | Autologous bone marrow mesenchymal stromal cells | - | 12 |

(-) Not stated, unclear, or unable to ascertain; IRQ: Interquartile range; *Author-developed measures were reported with author’s name; ** The name of questionnaire was extracted from the protocol, since it was not clearly stated in the primary publication (only “Pain questionnaire” was reported); # Abstract; ^ Data reported only for patients with ADPKD; # Abstract; ! More than one studied referred to the same publication; ^¥^ Study duration was reported as mean months of follow-up.

^a^ Abdominal pain; ^b^ Abdominal and back pain; ^c^ Kidney pain; ^d^ Abdominal and epigastric pain (with or without abdominal distension and cramps); ^e^ At least one of the following: abdominal pain, abdominal distension, abdominal fullness, back pain; ^g^ Abdominal and chest pain; ^h^ Abdominal cramps, abdominal pain, headache and injection site pain; ^i^ Abdominal and flank pain; ^k^ Flank and back pain; ^l^ Epigastric, chest and injection site pain; ^m^ Abdominal, back, kidney, oropharyngeal pain and headache; ^n^ Abdominal, back, flank, precordial, muscular, site injection, neuropathic pain and headache; ^o^ Chest pain; ^p^ Abdominal, back or flank (kidney pain), joint pain and headache; ^q^ Abdominal, flank, musculoskeletal, and headache with or without genital pain; ^r^ Abdominal, back, flank and oropharyngeal pain and headache; ^s^ Beck and/or flank (kidney pain) pain and headache; ^t^ Abdominal, back, flank, kidney, musculoskeletal, oropharyngeal, neck and chest pain, pain in extremity, and headache; ^u^ VAS was specified by author that was an instrument specific for pain.

**References**

1. Braun M, Young J, Reiner CS, Poster D, Krauer F, Kistler AD, et al. Low-dose oral sirolimus and the risk of menstrual-cycle disturbances and ovarian cysts: analysis of the randomized controlled SUISSE ADPKD trial. PLoS ONE. 2012;7(10):e45868.

2. Caroli A, Perico N, Perna A, Antiga L, Brambilla P, Pisani A, et al. Effect of longacting somatostatin analogue on kidney and cyst growth in autosomal dominant polycystic kidney disease (ALADIN): A randomised, placebo-controlled, multicentre trial. The Lancet. 2013;382(9903):1485-95. doi: <http://dx.doi.org/10.1016/S0140-6736%2813%2961407-5>. PubMed PMID: 52741880.

3. Casteleijn NF, Blais JD, Chapman AB, Czerwiec FS, Devuyst O, Higashihara E, et al. Tolvaptan and Kidney Pain in Patients With Autosomal Dominant Polycystic Kidney Disease: Secondary Analysis From a Randomized Controlled Trial. Am J Kidney Dis. 2017;69(2):210-9. doi: <http://dx.doi.org/10.1053/j.ajkd.2016.08.028>. PubMed PMID: 613824576.

4. Chrispijn M, Gevers TJG, Hol JC, Monshouwer R, Dekker HM, Drenth JPH. Everolimus does not further reduce polycystic liver volume when added to long acting octreotide: Results from a randomized controlled trial. J Hepatol. 2013;59(1):153-9. doi: <http://dx.doi.org/10.1016/j.jhep.2013.03.004>. PubMed PMID: 52528110.

5. Chrispijn M, Nevens F, Gevers TJ, Vanslembrouck R, van Oijen MG, Coudyzer W, et al. The long-term outcome of patients with polycystic liver disease treated with lanreotide. Aliment Pharmacol Ther. 2012;35(2):266-74.

6. D'Agnolo HM, Kievit W, Takkenberg RB, Riano I, Bujanda L, Neijenhuis MK, et al. Ursodeoxycholic acid in advanced polycystic liver disease: A phase 2 multicenter randomized controlled trial. J Hepatol. 2016;65(3):601-7. doi: <https://dx.doi.org/10.1016/j.jhep.2016.05.009>. PubMed PMID: 27212247.

7. Davis S, Gralla J, Chan L, Wiseman A, Edelstein CL. Effect of Sirolimus on Native Total Kidney Volume After Transplantation in Patients with Autosomal Dominant Polycystic Kidney Disease: A Randomized Controlled Pilot Study. Transplant Proc. 2018;50(5):1243-8. doi: <https://dx.doi.org/10.1016/j.transproceed.2018.02.060>. PubMed PMID: 29880342.

8. Devuyst O, Chapman AB, Gansevoort RT, Higashihara E, Perrone RD, Torres VE, et al. Urine Osmolality, Response to Tolvaptan, and Outcome in Autosomal Dominant Polycystic Kidney Disease: Results from the TEMPO 3:4 Trial. J Am Soc Nephrol. 2017;28(5):1592-602. doi: <https://dx.doi.org/10.1681/ASN.2016040448>. PubMed PMID: 27920153.

9. El-Damanawi R, Lee M, Harris T, Cowley LB, Bond S, Pavey H, et al. High Water versus Ad libitum Water Intake for Autosomal Dominant Polycystic Kidney Disease: A Randomised Controlled Feasibility Trial. Qjm. 2019;30:30. doi: <https://dx.doi.org/10.1093/qjmed/hcz278>. PubMed PMID: 31665476.

10. Eudra CD. A multi-center, open-label, extension study to evaluate the long-term efficacy and safety of oral tolvaptan tablet regimens in subjects with autosomal dominant polycystic kidney disease (ADPKD). <https://wwwclinicaltrialsregistereu/ctr-search/trial/2010-018401-10/DE>. 2015.

11. Gansevoort RT, van Gastel MDA, Chapman AB, Blais JD, Czerwiec FS, Higashihara E, et al. Plasma copeptin levels predict disease progression and tolvaptan efficacy in autosomal dominant polycystic kidney disease. Kidney Int. 2019;96(1):159-69. doi: <http://dx.doi.org/10.1016/j.kint.2018.11.044>. PubMed PMID: 2001701977.

12. Hogan MC, Masyuk TV, Page L, Holmes DR, Li X, Bergstralh EJ, et al. Somatostatin analog therapy for severe polycystic liver disease: Results after 2 years. Nephrol Dial Transplant. 2012;27(9):3532-9. doi: <http://dx.doi.org/10.1093/ndt/gfs152>. PubMed PMID: 365568852.

13. Hogan MC, Masyuk TV, Page LJ, Kubly VJ, Bergstralh EJ, Li X, et al. Randomized clinical trial of long-acting somatostatin for autosomal dominant polycystic kidney and liver disease. J Am Soc Nephrol. 2010;21(6):1052-61. doi: <https://dx.doi.org/10.1681/ASN.2009121291>. PubMed PMID: 20431041.

14. Irazabal MV, Blais JD, Perrone RD, Gansevoort RT, Chapman AB, Devuyst O, et al. Prognostic Enrichment Design in Clinical Trials for Autosomal Dominant Polycystic Kidney Disease: The TEMPO 3:4 Clinical Trial. KI Rep. 2016;1(4):213-20. doi: <https://dx.doi.org/10.1016/j.ekir.2016.08.001>. PubMed PMID: 29142926.

15. Meijer E, Visser FW, Van Aerts RMM, Blijdorp CJ, Casteleijn NF, D'Agnolo HMA, et al. Effect of lanreotide on kidney function in patients with autosomal dominant polycystic kidney disease the DIPAK 1 randomized clinical trial. JAMA - Journal of the American Medical Association. 2018;320(19):2010-9. doi: <http://dx.doi.org/10.1001/jama.2018.15870>. PubMed PMID: 624636781.

16. Muto S, Kawano H, Higashihara E, Narita I, Ubara Y, Matsuzaki T, et al. The effect of tolvaptan on autosomal dominant polycystic kidney disease patients: a subgroup analysis of the Japanese patient subset from TEMPO 3:4 trial. Clin Exp Nephrol. 2015;19(5):867-77. doi: <https://dx.doi.org/10.1007/s10157-015-1086-2>. PubMed PMID: 25663351.

17. Muto S, Okada T, Yasuda M, Tsubouchi H, Nakajima K, Horie S. Long-term safety profile of tolvaptan in autosomal dominant polycystic kidney disease patients: TEMPO Extension Japan Trial. Drug healthc. 2017;9:93-104. doi: <https://dx.doi.org/10.2147/DHPS.S142825>. PubMed PMID: 29123425.

18. Perico N, Ruggenenti P, Perna A, Caroli A, Trillini M, Sironi S, et al. Octreotide-LAR in later-stage autosomal dominant polycystic kidney disease (ALADIN 2): A randomized, double-blind, placebo-controlled, multicenter trial. PLoS Med. 2019;16(4):e1002777. doi: <https://dx.doi.org/10.1371/journal.pmed.1002777>. PubMed PMID: 30951521.

19. Ruggenenti P, Gentile G, Perico N, Perna A, Barcella L, Trillini M, et al. Effect of Sirolimus on Disease Progression in Patients with Autosomal Dominant Polycystic Kidney Disease and CKD Stages 3b-4. Clin J Am Soc Nephrol. 2016;11(5):785-94. doi: <https://dx.doi.org/10.2215/CJN.09900915>. PubMed PMID: 26912555.

20. Schrier RW, Abebe KZ, Perrone RD, Torres VE, Braun WE, Steinman TI, et al. Blood pressure in early autosomal dominant polycystic kidney disease. N Engl J Med. 2014;371(24):2255-66. doi: <https://dx.doi.org/10.1056/NEJMoa1402685>. PubMed PMID: 25399733.

21. Serra AL, Kistler AD, Poster D, Krauer F, Senn O, Raina S, et al. Safety and tolerability of sirolimus treatment in patients with autosomal dominant polycystic kidney disease. Nephrol Dial Transplant. 2009;24(11):3334-42. doi: <https://dx.doi.org/10.1093/ndt/gfp280>. PubMed PMID: 19525519.

22. Serra AL, Poster D, Kistler AD, Krauer F, Raina S, Young J, et al. Sirolimus and kidney growth in autosomal dominant polycystic kidney disease. N Engl J Med. 2010;363(9):820-9. doi: <https://dx.doi.org/10.1056/NEJMoa0907419>. PubMed PMID: 20581391.

23. Tesar V, Ciechanowski K, Pei Y, Barash I, Shannon M, Li R, et al. Bosutinib versus Placebo for Autosomal Dominant Polycystic Kidney Disease. J Am Soc Nephrol. 2017;28(11):3404-13. doi: <https://dx.doi.org/10.1681/ASN.2016111232>. PubMed PMID: 28838955.

24. Torres VE, Abebe KZ, Chapman AB, Schrier RW, Braun WE, Steinman TI, et al. Angiotensin blockade in late autosomal dominant polycystic kidney disease. N Engl J Med. 2014;371(24):2267-76. doi: <http://dx.doi.org/10.1056/NEJMoa1402686>. PubMed PMID: 600807626.

25. Torres VE, Chapman AB, Devuyst O, Gansevoort RT, Grantham JJ, Higashihara E, et al. Tolvaptan in patients with autosomal dominant polycystic kidney disease. N Engl J Med. 2012;367(25):2407-18. doi: <http://dx.doi.org/10.1056/NEJMoa1205511>. PubMed PMID: 366288790.

26. Torres VE, Chapman AB, Devuyst O, Gansevoort RT, Perrone RD, Dandurand A, et al. Multicenter, open-label, extension trial to evaluate the long-term efficacy and safety of early versus delayed treatment with tolvaptan in autosomal dominant polycystic kidney disease: the TEMPO 4:4 Trial. Nephrol Dial Transplant. 2018;33(3):477-89.

27. Torres VE, Chapman AB, Devuyst O, Gansevoort RT, Perrone RD, Koch G, et al. Tolvaptan in later-stage autosomal dominant polycystic kidney disease. N Engl J Med. 2017;377(20):1930-42. doi: <http://dx.doi.org/10.1056/NEJMoa1710030>. PubMed PMID: 619258302.

28. Torres VE, Higashihara E, Devuyst O, Chapman AB, Gansevoort RT, Grantham JJ, et al. Effect of tolvaptan in autosomal dominant polycystic kidney disease by CKD stage: Results from the TEMPO 3:4 trial. Clinical Journal of the American Society of Nephrology. 2016;11(5):803-11. doi: <http://dx.doi.org/10.2215/CJN.06300615>. PubMed PMID: 617127266.

29. Torres VE, Meijer E, Bae KT, Chapman AB, Devuyst O, Gansevoort RT, et al. Rationale and design of the TEMPO (Tolvaptan Efficacy and Safety in Management of Autosomal Dominant Polycystic Kidney Disease and its Outcomes) 3-4 Study. Am J Kidney Dis. 2011;57(5):692-9. doi: <https://dx.doi.org/10.1053/j.ajkd.2010.11.029>. PubMed PMID: 21333426.

30. van Aerts RMM, Kievit W, D'Agnolo HMA, Blijdorp CJ, Casteleijn NF, Dekker SEI, et al. Lanreotide Reduces Liver Growth In Patients With Autosomal Dominant Polycystic Liver and Kidney Disease. Gastroenterology. 2019;157(2):481-91.e7. doi: <https://dx.doi.org/10.1053/j.gastro.2019.04.018>. PubMed PMID: 31022403.

31. van KL, Nevens F, Vanslembrouck R, Oijen MG, Hoffmann AL, Dekker HM, et al. Lanreotide reduces the volume of polycystic liver: a randomized, double-blind, placebo-controlled trial. Gastroenterology. 2009;137(5):1661-8.

32. Walz G, Budde K, Mannaa M, Nurnberger J, Wanner C, Sommerer C, et al. Everolimus in patients with autosomal dominant polycystic kidney disease. N Engl J Med. 2010;363(9):830-40. doi: <https://dx.doi.org/10.1056/NEJMoa1003491>. PubMed PMID: 20581392.

33. Brioni E, Magnaghi C, Delli Zotti GB, Sangiovanni E, Sciarrone Alibrandi MT, Apuzzo L, et al. Valutazione Del Benessere Psico-Fisico Nell'aderenza Terapeutica Nelle Donne Con Malattia Renale Policistica Autosomica Dominante: Uno Studio Osservazionale. Giornale di Tecniche Nefrologiche e Dialitiche. 2019;31(3):160-6. doi: <http://dx.doi.org/10.1177/0394936219858903>. PubMed PMID: 628481301.

34. Kucuk EV, Tahra A, Bindayi A, Suceken FY, Onol FF, Boylu U. Long-term functional results of aspiration and sclerotherapy with ethanol in patients with autosomal dominant polycystic kidney disease: a non-randomized pilot clinical study. Int Urol Nephrol. 2016;48(4):457-63. doi: <https://dx.doi.org/10.1007/s11255-015-1211-x>. PubMed PMID: 26759329.

35. Lai S, Petramala L, Muscaritoli M, Cianci R, Mazzaferro S, Mitterhofer AP, et al. alpha-lipoic acid in patients with autosomal dominant polycystic kidney disease. Nutrition. 2020;71:110594. doi: <https://dx.doi.org/10.1016/j.nut.2019.110594>. PubMed PMID: 31790890.

36. Makhlough A, Shekarchian S, Moghadasali R, Einollahi B, Hosseini SE, Jaroughi N, et al. Safety and tolerability of autologous bone marrow mesenchymal stromal cells in ADPKD patients. Stem Cell Res Ther. 2017;8(1):116. doi: <https://dx.doi.org/10.1186/s13287-017-0557-7>. PubMed PMID: 28535817.
