# Supplementary material for: Patient-reported outcome measures for pain in autosomal dominant polycystic kidney disease: A systematic review
Source: PLoS One. 2021 May 27;16(5):e0252479. doi: 10.1371/journal.pone.0252479 (PMC8158964; doi:10.1371/journal.pone.0252479)
Supplement: S3 Table — (DOCX) [file pone.0252479.s003.docx]

**S3 Table. Characteristics of the non-interventional studies**

| **Study ID** | **Publication year** | **Country** | **Sample size** | **Type of intervention (if applicable)** | **Measure used to assess pain*** | **Study duration (months) ^¥^** |
| --- | --- | --- | --- | --- | --- | --- |
| **Retrospective study** | | | | | | |
| **Abraham 2015[1] ^a^** | 2015 | India | 75 | Laparoscopic nephrectomy | Abraham 2015[1] | 120 |
| **Bistritz 2005[2] ^a^** | 2005 | Canada | 38^ | - | - | - |
| **Brown 1996[3] ^c^**  **(SUISSE ADPKD study)** | 1996 | USA | 8^ | Laparoscopic cyst marsupialization |  | - |
| **Chiu 2007[4] ^a^** | 2007 | United Kingdom | 158 | - | - | 84 |
| **Daineko 2019[5] ^e^** | 2019 | Russia | 46 | Kidney transplantation with or without previous nephrectomy | - | 43 |
| **Delaney 1985[6] ^g^** | 1985 | USA | 53 | - | - | 144 |
| **Desai 2008[7] ^h^** | 2008 | USA | 12 | Bilateral laparoscopic nephrectomy | - | 0.1 |
| **Dunn 2000[8] ^i^** | 2000 | USA | 9 | Laparoscopic nephrectomies | VAS ^°^ | 31 |
| **Dunn 2001[9] ^j^** | 2001 | USA | 15 | Laparoscopic cyst marsupialization | VAS ^°^ | 26 |
| **Fary 2010[10] ^c^** | 2010 | Africa | 55 | - | - | 120 |
| **Game 2003[11]** | 2003 | France | 6 | Laparoscopic nephrectomy | - | 14 |
| **Iijima 2017[12]** | 2017 | Japan | 11^ | Minocycline hydrochloride cyst aspiration sclerotherapy | - | 12 |
| **Jenkins 2002[13] ^i^** | 2002 | USA | 4 | Bilateral laparoscopic nephrectomy | - | 0.5 |
| **Jouret 2011[14] ^n^** | 2011 | Belgium | 24 | - | - | 18 |
| **Kim 2015[15] ^o^** | 2015 | Korea | 461 | - | GSRS (Revised) | - |
| **Kim 2009[16] ^p^** | 2009 | Korea | 21 | Cyst ablation N-butyl cyanoacrylate and iodized oil | - | 54 |
| **Levine 1981[17] ^g^** | 1981 | USA | 17^ | - | - | - |
| **Levine 1985[18] ^i^** | 1985 | USA | 35 | - | - | - |
| **Lipke 2007[19]** | 2007 | USA | 18 | Bilateral hand assisted laparoscopic nephrectomy | VAS ^°^ | 14 |
| **Lugnane 1990[20]** | 1990 | France | 54 | Kidney transplant | - | 60 |
| **Mandelssohn 1988[21] ^s^** | 1988 | Canada | 35 | - | - | 120 |
| **Petitpierre 2015[22] ^v^** | 2015 | France | 73 | Embolization of renal arteries | - | 26 |
| **Qian 2015[23]** | 2015 | China | 70 | Decortication | VAS | 62 |
| **Sallee 2009[24] ^x^** | 2009 | France | 36 | - | - | - |
| **Suwabe 2016[25] ^i^** | 2016 | Japan | 449 | Renal trans catheter arterial embolization | - | 12 |
| **Teichman 1995[26]** | 1995 | USA | 6 | Laparoscopic cyst marsupialization | - | - |
| **Wetzel 1993[27] ^ç^** | 1993 | France | 217 | - | - | 360 |
| **Whitten 2006[28] ^a^** | 2006 | USA | 10 | Bilateral hand-assisted laparoscopic nephrectomy |  | 3 |
| **Yu 2018[29] ^$^** | 2018 | China | 135 | Laparoscopic unilateral cyst decortications | VAS ^°^ | 12 |
| **Case-report** |  |  |  |  |  |  |
| **Akihisa 2018[30] ^b^** | 2018 | Japan | 1 | Living donor liver transplantation alone | - | - |
| **Eng 2008[31]** | 2008 | USA | 1 | Transplant from death ADPKD, during the follow up the patients performed bilateral laparoscopic native nephrectomies | - | 180 |
| **Ganpule 2016[32] ^l^** | 2016 | India | 1 | - | - | - |
| **Ghanbarinia 2008[33] ^h^** | 2008 | USA | 4 | - | - | - |
| **Hemal 1999[34] ^c^** | 1999 | India | 2 | Retroperitoneoscopic decortication | - | 12 |
| **Lantinga 2017[35] ^i!^**  **(DIPAK-1 study)** | 2017 | The Netherlands | 7 | Lanreotide (somatostatin analog) + standard care vs standard care | - | 30 |
| **Schelenz 2003[36] ^y^** | 2003 | United Kingdom | 2 | - | - | - |
| **Schwab 1983[37] ^i^** | 1983 | USA | 1 | Gentamicin + clindamycin | - | 2 |
| **Sedlacek 2008[38] ^i^** | 2008 | Lebanon | 1 | Posaconazole Salvage after failure with liposomal amphotericin | - | 28 |
| **Walsh 2012[39] ^§^** | 2012 | USA | 1 | Sequential celiac plexus blockade, radiofrequency ablation, and spinal cord stimulation | Walsh 2012[39] | 27 |
| **Case series** |  |  |  |  |  |  |
| **Akinci 2008[40] ^c^** | 2008 | Turkey | 3 | Percutaneous drainage and catheterization | - | 18 |
| **Bourquia 1989[41] ^b^** | 1989 | Morocco | 21 | - | - | 60 |
| **Mussche 1975[42]** | 1975 | Belgium | 81 | - | - | 120 |
| **Cohort** |  |  |  |  |  |  |
| **Cole 2013[43] ^#^** | 2013 | USA | 702 | - | ADPKD-IS | - |
| **de Jager 2018[44] ^d^** | 2018 | The Netherlands | 5^ | Percutaneous catheter-based renal denervation (RDN) (analgesic medication) | MPQ-DV | 12 |
| **Delakas 1997[45] ^f^** | 1997 | Greece | 13 | Extracorporeal shockwave lithotripsy (SWL) | - | 5 |
| **Delli Zotti 2019[46]** | 2019 | Italy | 37 | - | KDQOL-SF | 9 |
| **Elzinga 1992[47]** | 1992 | USA | 30 | Surgical cyst decompression | - | 21 |
| **Gevers 2015[48] ^b!^ (RESOLVE trial)** | 2015 | The Netherlands | 43 | Lanreotide (somatostatin analog) | EQ-5D; GI-Q | 6 |
| **Gevers 2014[49] ^b!#^ (RESOLVE trial)** | 2014 | The Netherlands | 43 | Lanreotide (somatostatin analog) | EQ-5D | 6 |
| **Gustafsson 2003[50]** | 2003 | Sweden | 6 | Liver transplantation | - | - |
| **Haseebuddin 2012[51]** | 2012 | USA | 19 | Laparoscopic Cyst Decortication | Haseebuddin 2012[51] | 131 |
| **He 2007[52] ^c^** | 2007 | China | 72 | Unilateral and bilateral cyst decompression | - | 60 |
| **Iliuta 2019[53] ^m^** | 2019 | Canada | 66 | Foam sclerotherapy | Iliuta 2019[53] | 17 |
| **Kirchner 2006[54]** | 2006 | Germany | 29^ | Liver or liver-kidney transplantation | SF-36 | 62 |
| **Krol 2006[55] ^i^** | 2006 | Poland | 18^ | Simultaneous bilateral trans peritoneal nephrectomy | - | - |
| **Lee 2003_1[56] ^q^** | 2003 | USA | 29 | Laparoscopic cyst decortication | VAS ^°^; SF-36 | 32 |
| **Lee 2004[57] ^c^** | 2004 | USA | 3 | Hand-assisted laparoscopic bilateral nephrectomy | VAS ^°^ | - |
| **Lee 2003_2[58] ^i^** | 2003 | Korea | 11 | Absolute ethanol cyst ablation | - | 12 |
| **Liu 2012[59] ^r^** | 2012 | China | 13 | Flexible ureteroscopy and holmium laser lithotripsy | - | 41 |
| **Rehman 2001[60] ^w^** | 2001 | Brazil | 3 | Laparoscopic nephrectomy | VAS ^°^ | 7 |
| **Rizk 2009[61]**  **(COHORT study)** | 2009 | USA | 152 | - | SF-36 | 1 |
| **Sakuhara 2015[62] ^o^** | 2015 | Japan | 15 | Trans catheter arterial embolization with ethanol injection | Sakuhara 2015[62] | 24 |
| **Seshadri 2001[63]** | 2001 | Canada | 10 | Laparoscopic nephrectomy | - | 12 |
| **Struthers 1997[64] ^c^** | 1997 | Canada | 10 | Percutaneous cyst aspiration | - | 1 |
| **Sulikowski 2006[65] ^x^** | 2006 | Poland | 30 | Laparoscopic cyst excisions or nephrectomy | VAS ^°^ | 12 |
| **Sulikowski 2009[66] ^e^** | 2009 | Poland | 183 | Kidney transplantation | - | 84 |
| **Suwabe 2017[67] ^c^** | 2017 | Japan | 188 | Renal trans catheter arterial embolization | SF-36;  Suwabe 2017[67] | 12 |
| **Case-control** |  |  |  |  |  |  |
| **Christophe 1996[68] ^d^** | 1996 | Belgium | 50^ | - | - | 48 |
| **Fitzpatrick 1990[69] ^k^** | 1990 | USA | 54^ | Kidney transplantation | - | 36 |
| **Ishikawa 1996[70] ^a^** | 1996 | Japan | 55^ | - | - | - |
| **Jacquet 2011[71]** | 2011 | France | 534^ | Kidney transplantation |  | 180 |
| **Lifson 1998[72]** | 1998 | USA | 8^ | Laparoscopic cyst decortication | - | 24 |
| **Morino 1994[73] ^a^** | 1994 | Italy | 7^ | Laparoscopic fenestration | - | - |
| **Neijenhuis 2016[74] ^u^** | 2016 | The Netherlands and USA | 228^ | - | PLD-Q; EORTC QLQ-C30; EQ-5D | 0.5 |
| **Timio 1992[75] ^z^** | 1992 | Italy | - | - | - | 120 |
| **Cross-sectional** |  |  |  |  |  |  |
| **D’Agnolo 2016[76] ^£#!^**  **(DIPAK-1 study)** | 2016 | The Netherlands | 309 | Lanreotide (somatostatin analog) + standard care vs standard care | GI-Q;  D’Agnolo 2016[76] ^#!^ | - |
| **D’Agnolo 2017[77] ^£!^**  **(DIPAK-1 study)** | 2017 | The Netherlands | 309 | Lanreotide (somatostatin analog) + standard care vs standard care | GI-Q;  D’Agnolo 2017[77] ^!^ | - |
| **Eriksson 2017[78] ^d^** | 2017 | Denmark, Finland, Norway and Sweden | 243 | - | EQ-5D; SF-12 | - |
| **Hogan 2015[79] ^!^**  **(HALT-PKD-A study)** | 2015 | USA | 558 | Lisinopril (angiotensin I–converting enzyme inhibitor) + telmisartan (angiotensin II–receptor blocker) vs Lisinopril + placebo | SF-36 | 96 |
| **Miskulin 2014[80] ^t!^**  **(HALT-PKD study)** | 2014 | USA | 1043 | Lisinopril (angiotensin I–converting enzyme inhibitor) + telmisartan (angiotensin II–receptor blocker) vs Lisinopril + placebo | SF-36; Wisconsin BPS (Revised) | - |
| **Suwabe 2013[81]** | 2013 | Japan | 219 | - | SF-36;  Suwabe 2013[81] | - |
| **Taylor 2005[82] ^o^** | 2005 | USA | 637 | - | Taylor 2005[82] | - |

(-) Not stated, unclear, or unable to ascertain; SD: Standard deviations; RCT: Randomized Controlled Trial; *Author-developed measures were reported with author’s name; ^ Data reported only for patients with ADPKD; # Abstract; ! More than one studied referred to the same publication; ^¥^ Study duration was reported as mean months of follow-up.

^a^ Abdominal pain; ^b^ Abdominal and/or back pain; ^c^ Flank pain; ^d^ Kidney pain; ^e^ Lumbar pain; ^f^ Colic pain; ^g^ Abdominal, flank and lumbar pain with or without colic; ^h^ Abdominal pain (inclusion criteria) and chest pain (outcome); ^i^ Abdominal and/or flank pain with or without cramps; ^j^ Abdominal, flank, groin pain and headache; ^k^ Abdominal pain and headache; ^l^ Painful cervical lymphadenopathy in the neck; ^m^ Abdominal, flank, back pain and abdominal distension; ^n^ Abdominal, epigastric and loin pain; ^o^ Abdominal, back and flank pain; ^p^ Abdominal distension and/or pain; ^q^ Abdominal and flank pain and headache; ^r^ Flank and stent pain; ^s^ Abdominal pain and headache; ^t^ Abdominal, back and radicular pain; ^u^ Back, rib cage, stomach and in side pain; ^v^ Lumbar flank and kidney pain; ^w^ Flank, back and incisional pain; ^x^ Abdominal and/or lumbar pain; ^y^ Abdominal, peri-orbital, ocular pain and headache; ^z^ Chest pain; ^§^ Abdominal, flank, lumbar and epigastric pain; ^$^ Kidney and flank pain; ^£^ Kidney and liver pain (renal pain was defined as pain or discomfort located in the flank, the lower back or abdomen to patients was caused by renal cysts. Liver pain was defined as pain or discomfort located in the right upper abdomen, behind or below the rib cage that according to patients’ well-being is most likely to be caused by liver cysts; ^ç^ Back and lumbar pain; ^°^ VAS was specified by author that was an instrument specific for pain.

**References**

1. Abraham GP, Siddaiah AT, Das K, Ramaswami K, George DP, Thampan OS. Laparoscopic nephrectomy for autosomal dominant polycystic kidneys in patients with end-stage renal disease on maintenance hemodialysis: 10-year single surgeon experience from an Indian center. J. 2015;11(3):187-92. doi: <https://dx.doi.org/10.4103/0972-9941.140217>. PubMed PMID: 26195877.

2. Bistritz L, Tamboli C, Bigam D, Bain VG. Polycystic liver disease: experience at a teaching hospital. Am J Gastroenterol. 2005;100(10):2212-7. PubMed PMID: 16181371.

3. Brown JA, Torres VE, King BF, Segura JW. Laparoscopic marsupialization of symptomatic polycystic kidney disease. J Urol. 1996;156(1):22-7. PubMed PMID: 8648810.

4. Chiu DYY, Whiteside AM, Hegarty J, Wood G, O'Donoghue DJ, Waldek S, et al. Epidemiology and investigation of acute abdominal presentations in autosomal dominant polycystic kidney disease [7]. Nephrol Dial Transplant. 2007;22(5):1483-4. doi: <http://dx.doi.org/10.1093/ndt/gfl827>. PubMed PMID: 47072249.

5. Daineko VS, Ananiev AN, Nevirovich ES, Skvorcov AE, Budylev SA, Selivanov AN, et al. Results of kidney transplantation in patients with end-stage renal failure caused by autosomal dominant polycystic kidney disease. [Russian]. Vestnik Transplantologii i Iskusstvennykh Organov. 2019;21(2):39-48. doi: <http://dx.doi.org/10.15825/1995-1191-2019-2-39-48>. PubMed PMID: 2002507843.

6. Delaney VB, Adler S, Bruns FJ, Licinia M, Segel DP, Fraley DS. Autosomal dominant polycystic kidney disease: presentation, complications, and prognosis. Am J Kidney Dis. 1985;5(2):104-11. PubMed PMID: 3970015.

7. Desai PJ, Castle EP, Daley SM, Swanson SK, Ferrigni RG, Humphreys MR, et al. Bilateral laparoscopic nephrectomy for significantly enlarged polycystic kidneys: a technique to optimize outcome in the largest of specimens. BJU Int. 2008;101(8):1019-23. doi: <https://dx.doi.org/10.1111/j.1464-410X.2007.07423.x>. PubMed PMID: 18190626.

8. Dunn MD, Portis AJ, Elbahnasy AM, Shalhav AL, Rothstein M, McDougall EM, et al. Laparoscopic nephrectomy in patients with end-stage renal disease and autosomal dominant polycystic kidney disease. Am J Kidney Dis. 2000;35(4):720-5. PubMed PMID: 10739795.

9. Dunn MD, Portis AJ, Naughton C, Shalhav A, McDougall EM, Clayman RV. Laparoscopic cyst marsupialization in patients with autosomal dominant polycystic kidney disease. J Urol. 2001;165(6 Pt 1):1888-92. PubMed PMID: 11371874.

10. Fary Ka E, Seck SM, Niang A, Cisse MM, Diouf B. Patterns of autosomal dominant polycystic kidney diseases in black Africans. Saudi J Kidney Dis Transpl. 2010;21(1):81-6. PubMed PMID: 20061698.

11. Game X, Vaessen C, Mouzin M, Mallet R, Malavaud B, Sarramon JP, et al. Retroperitoneal laparoscopic nephrectomy fo polycystic kidney: preliminary results. [French]. Progres en urologie : journal de l'Association francaise d'urologie et de la Societe francaise d'urologie. 2003;13(2):215-21. PubMed PMID: 36911933.

12. Iijima T, Suwabe T, Sumida K, Hayami N, Mise K, Hoshino J, et al. Prediction of hepatic cyst recurrence after minocycline hydrochloride aspiration sclerotherapy using cyst computed tomography values. Hepatology Research. 2017;47(5):419-24. doi: <http://dx.doi.org/10.1111/hepr.12763>. PubMed PMID: 611404039.

13. Jenkins MA, Crane JJ, Munch LC. Bilateral hand-assisted laparoscopic nephrectomy for autosomal dominant polycystic kidney disease using a single midline HandPort incision. Urology. 2002;59(1):32-6. PubMed PMID: 11796276.

14. Jouret F, Lhommel R, Beguin C, Devuyst O, Pirson Y, Hassoun Z, et al. Positron-emission computed tomography in cyst infection diagnosis in patients with autosomal dominant polycystic kidney disease. Clin J Am Soc Nephrol. 2011;6(7):1644-50. doi: <https://dx.doi.org/10.2215/CJN.06900810>. PubMed PMID: 21700816.

15. Kim H, Park HC, Ryu H, Kim K, Kim HS, Oh KH, et al. Clinical Correlates of Mass Effect in Autosomal Dominant Polycystic Kidney Disease. PLoS ONE. 2015;10(12):e0144526. doi: <https://dx.doi.org/10.1371/journal.pone.0144526>. PubMed PMID: 26641645.

16. Kim SH, Kim SH, Cho JY. Cyst ablation using a mixture of N-butyl cyanoacrylate and iodized oil in patients with autosomal dominant polycystic kidney disease: the long-term results. Korean J Radiol. 2009;10(4):377-83. doi: <https://dx.doi.org/10.3348/kjr.2009.10.4.377>. PubMed PMID: 19568466.

17. Levine E, Grantham JJ. The role of computed tomography in the evaluation of adult polycystic kidney disease. Am J Kidney Dis. 1981;1(2):99-105. PubMed PMID: 7332005.

18. Levine E, Grantham JJ. High-density renal cysts in autosomal dominant polycystic kidney disease demonstrated by CT. Radiology. 1985;154(2):477-82. PubMed PMID: 3966136.

19. Lipke MC, Bargman V, Milgrom M, Sundaram CP. Limitations of laparoscopy for bilateral nephrectomy for autosomal dominant polycystic kidney disease. J Urol. 2007;177(2):627-31. PubMed PMID: 17222647.

20. Lugagne PM, Hiesse C, Bellamy J, Charpentier E, Bensadoun H, Fries D, et al. [Autosomal dominant polycystic kidney in adults and transplantation]. Ann Urol (Paris). 1990;24(4):272-7. PubMed PMID: 2221829.

21. Mendelssohn DC, Harding ME, Cardella CJ, Cook GT, Uldall PR. Management of end-stage autosomal dominant polycystic kidney disease with hemodialysis and transplantation. Clin Nephrol. 1988;30(6):315-9. PubMed PMID: 3072137.

22. Petitpierre F, Cornelis F, Couzi L, Lasserre AS, Tricaud E, Le Bras Y, et al. Embolization of renal arteries before transplantation in patients with polycystic kidney disease: a single institution long-term experience. Eur Radiol. 2015;25(11):3263-71. doi: <https://dx.doi.org/10.1007/s00330-015-3730-3>. PubMed PMID: 25981217.

23. Qian X, Sheng X, Li R, Liu H, Kong X, Duan L, et al. Which Stage of ADPKD Is More Appropriate for Decortication? A Retrospective Study of 137 Patients from a Single Clinic. PLoS ONE. 2015;10(5):e0120696. doi: <https://dx.doi.org/10.1371/journal.pone.0120696>. PubMed PMID: 25939015.

24. Sallee M, Rafat C, Zahar JR, Paulmier B, Grunfeld JP, Knebelmann B, et al. Cyst infections in patients with autosomal dominant polycystic kidney disease. Clin J Am Soc Nephrol. 2009;4(7):1183-9. doi: <https://dx.doi.org/10.2215/CJN.01870309>. PubMed PMID: 19470662.

25. Suwabe T, Ubara Y, Mise K, Ueno T, Sumida K, Yamanouchi M, et al. Suitability of Patients with Autosomal Dominant Polycystic Kidney Disease for Renal Transcatheter Arterial Embolization. J Am Soc Nephrol. 2016;27(7):2177-87. doi: <https://dx.doi.org/10.1681/ASN.2015010067>. PubMed PMID: 26620095.

26. Teichman JM, Hulbert JC. Laparoscopic marsupialization of the painful polycystic kidney. J Urol. 1995;153(4):1105-7. PubMed PMID: 7869473.

27. Wetzel O, Hormi M, Le Normand L, Karam G, Guenel J, Auvigne J, et al. Autosomal dominant polycystic kidney disease: urologic complications and results of kidney transplantation: 217 patients. [French]. Progres en urologie : journal de l'Association francaise d'urologie et de la Societe francaise d'urologie. 1993;3(2):252-62. PubMed PMID: 23830965.

28. Whitten MG, Van der Werf W, Belnap L. A novel approach to bilateral hand-assisted laparoscopic nephrectomy for autosomal dominant polycystic kidney disease. Surg Endosc. 2006;20(4):679-84. PubMed PMID: 16432653.

29. Yu J, Li B, Xiang YZ, Qi TG, Jin XB, Xiong H. Should kidney volume be used as an indicator of surgical occasion for patients with autosomal dominant polycystic kidney disease? Medicine (Baltimore). 2018;97(27):e11445. doi: <https://dx.doi.org/10.1097/MD.0000000000011445>. PubMed PMID: 29979446.

30. Akihisa T, Ino A, Egawa H, Kotera Y, Ariizumi S, Oomori A, et al. A case of a maintenance hemodialysis patient with autosomal dominant polycystic kidney disease who underwent living donor liver transplantation alone due to refractory liver cyst infection. CEN Case Rep. 2018;7(2):307-12. doi: <http://dx.doi.org/10.1007/s13730-018-0348-8>. PubMed PMID: 624281783.

31. Eng MK, Zorn KC, Harland RC, Bernstein AJ, Katz M, Shikanov S, et al. Fifteen-year follow-up of transplantation of a cadaveric polycystic kidney: a case report. Transplant Proc. 2008;40(5):1747-50. doi: <https://dx.doi.org/10.1016/j.transproceed.2008.01.065>. PubMed PMID: 18589185.

32. Ganpule AP, Chabra JS, Singh AG, Tak GR, Soni S, Sabnis R, et al. Case Report: Kikuchi-Fujimoto disease: A diagnostic and therapeutic dilemma following pretransplant nephrectomy for a 2.35 Kg kidney [version 1; referees: 2 approved]. F1000Research. 2016;5 (no pagination)(1407). doi: <http://dx.doi.org/10.12688/F1000RESEARCH.8992.1>. PubMed PMID: 614209586.

33. Ghanbarinia A, Chandra S, Chhabra K, Jain D. Renal abnormalities as incidental findings on myocardial single photon emission computed tomography perfusion imaging. Nucl Med Commun. 2008;29(7):588-92. doi: <http://dx.doi.org/10.1097/MNM.0b013e3282f8148b>. PubMed PMID: 354695374.

34. Hemal AK, Gupta NP, Rajeev TP, Aron M, Bhowmik D, Jain R. Retroperitoneoscopic management of infected cysts in adult polycystic kidney disease. Urol Int. 1999;62(1):40-3. doi: <http://dx.doi.org/10.1159/000030354>. PubMed PMID: 29377927.

35. Lantinga MA, D'Agnolo HM, Casteleijn NF, de Fijter JW, Meijer E, Messchendorp AL, et al. Hepatic Cyst Infection During Use of the Somatostatin Analog Lanreotide in Autosomal Dominant Polycystic Kidney Disease: An Interim Analysis of the Randomized Open-Label Multicenter DIPAK-1 Study. Drug Saf. 2017;40(2):153-67. doi: <https://dx.doi.org/10.1007/s40264-016-0486-x>. PubMed PMID: 27995519.

36. Schelenz S, Goldsmith DJA. Aspergillus endophthalmitis: An unusual complication of disseminated infection in renal transplant patients. Journal of Infection. 2003;47(4):336-43. doi: <http://dx.doi.org/10.1016/S0163-4453%2803%2900078-1>. PubMed PMID: 37322055.

37. Schwab S, Hinthorn D, Diederich D, Cuppage F, Grantham J. pH-dependent accumulation of clindamycin in a polycystic kidney. Am J Kidney Dis. 1983;3(1):63-6. doi: <http://dx.doi.org/10.1016/S0272-6386(83)80012-2>. PubMed PMID: 13020452.

38. Sedlacek M, Cotter JG, Suriawinata AA, Kaneko TM, Zuckerman RA, Parsonnet J, et al. Mucormycosis Peritonitis: More Than 2 Years of Disease-Free Follow-up After Posaconazole Salvage Therapy After Failure of Liposomal Amphotericin B. Am J Kidney Dis. 2008;51(2):302-6. doi: <http://dx.doi.org/10.1053/j.ajkd.2007.09.026>. PubMed PMID: 351106916.

39. Walsh N, Sarria JE. Management of chronic pain in a patient with autosomal dominant polycystic kidney disease by sequential celiac plexus blockade, radiofrequency ablation, and spinal cord stimulation. Am J Kidney Dis. 2012;59(6):858-61. doi: <https://dx.doi.org/10.1053/j.ajkd.2011.12.018>. PubMed PMID: 22361041.

40. Akinci D, Turkbey B, Yilmaz R, Akpinar E, Ozmen MN, Akhan O. Percutaneous treatment of pyocystis in patients with autosomal dominant polycystic kidney disease. Cardiovasc Intervent Radiol. 2008;31(5):926-30. doi: <https://dx.doi.org/10.1007/s00270-007-9273-5>. PubMed PMID: 18196333.

41. Bourquia A, Ramdani B, Jabrane AJ, Khajij T, Zaid D. Polycystic kidney disease. [French]. Rev Med Interne. 1989;10(4):313-8. PubMed PMID: 19218875.

42. Mussche M, Casneuf R, Ringoir S. Polycystic kidney. Study of 81 adults (Dutch). [Dutch]. Tijdschrift voor Geneeskunde. 1975;31(21):1061-4. PubMed PMID: 6188996.

43. Cole JC, Cheng R, Oberdhan D, Krasa HB, Hays RD. Psychometric analyses of patient-reported outcome instruments for autosomal dominant polycystic kidney disease. Value in Health. 2013;16 (3):A183. doi: <http://dx.doi.org/10.1016/j.jval.2013.03.921>. PubMed PMID: 71060802.

44. de Jager RL, Casteleijn NF, de Beus E, Bots ML, Vonken EE, Gansevoort RT, et al. Catheter-based renal denervation as therapy for chronic severe kidney-related pain. Nephrol Dial Transplant. 2018;33(4):614-9. doi: <https://dx.doi.org/10.1093/ndt/gfx086>. PubMed PMID: 28645206.

45. Delakas D, Daskalopoulos G, Cranidis A. Extracorporeal shockwave lithotripsy for urinary calculi in autosomal dominant polycystic kidney disease. J Endourol. 1997;11(3):167-70. PubMed PMID: 9181443.

46. Delli Zotti GB, Sangiovanni E, Brioni E, Ratti MM, Sciarrone Aliprandi MT, Spotti D, et al. [Psychological Assessment of a sample of women with ADPKD: quality of life, body image, anxiety and depression]. G. 2019;36(2). PubMed PMID: 30983181.

47. Elzinga LW, Barry JM, Torres VE, Zincke H, Wahner HW, Swan S, et al. Cyst decompression surgery for autosomal dominant polycystic kidney disease. J Am Soc Nephrol. 1992;2(7):1219-26. PubMed PMID: 1591362.

48. Gevers TJ, Hol JC, Monshouwer R, Dekker HM, Wetzels JF, Drenth JP. Effect of lanreotide on polycystic liver and kidneys in autosomal dominant polycystic kidney disease: an observational trial. Liver Int. 2015;35(5):1607-14. doi: <https://dx.doi.org/10.1111/liv.12726>. PubMed PMID: 25369108.

49. Gevers TJG, Hol JC, Monshouwer R, Dekker HM, Wetzels JF, Drenth JP. Effect of lanreotide on polycystic liver and kidney growth in patients with autosomal dominant polycystic kidney disease: An observational trial. United European Gastroenterology Journal. 2014;1):A140. doi: <http://dx.doi.org/10.1177/2050640614548980>.

50. Gustafsson BI, Friman S, Mjornstedt L, Olausson M, Backman L. Liver transplantation for polycystic liver disease - Indications and outcome. Transplant Proc. 2003;35(2):813-4. doi: <http://dx.doi.org/10.1016/S0041-1345%2803%2900081-2>. PubMed PMID: 36338170.

51. Haseebuddin M, Tanagho YS, Millar M, Roytman T, Chen C, Clayman RV, et al. Long-term impact of laparoscopic cyst decortication on renal function, hypertension and pain control in patients with autosomal dominant polycystic kidney disease. J Urol. 2012;188(4):1239-44. doi: <https://dx.doi.org/10.1016/j.juro.2012.06.026>. PubMed PMID: 22902029.

52. He ZZ, Song XS. Clinical effect and rationality of cyst decompression for adult polycystic kidney disease patients. [Chinese]. Journal of Dalian Medical University. 2007;29(3):265-7. PubMed PMID: 46951621.

53. Iliuta IA, Shi B, Pourafkari M, Akbari P, Bruni G, Hsiao R, et al. Foam Sclerotherapy for Cyst Volume Reduction in Autosomal Dominant Polycystic Kidney Disease: A Prospective Cohort Study. Kidney Medicine. 2019;1(6):366-75. doi: <http://dx.doi.org/10.1016/j.xkme.2019.07.015>. PubMed PMID: 2003757533.

54. Kirchner GI, Rifai K, Cantz T, Nashan B, Terkamp C, Becker T, et al. Outcome and quality of life in patients with polycystic liver disease after liver or combined liver-kidney transplantation. Liver Transpl. 2006;12(8):1268-77. doi: <http://dx.doi.org/10.1002/lt.20780>. PubMed PMID: 44194596.

55. Krol R, Ziaja J, Cierniak T, Pawlicki J, Chudek J, Wiecek A, et al. Simultaneous transabdominal bilateral nephrectomy in potential kidney transplant recipients. Transplant Proc. 2006;38(1):28-30. doi: <http://dx.doi.org/10.1016/j.transproceed.2005.12.099>. PubMed PMID: 43303590.

56. Lee DI, Andreoni CR, Rehman J, Landman J, Ragab M, Yan Y, et al. Laparoscopic cyst decortication in autosomal dominant polycystic kidney disease: impact on pain, hypertension, and renal function. J Endourol. 2003;17(6):345-54. PubMed PMID: 12965058.

57. Lee DI, Clayman RV. Hand-assisted laparoscopic nephrectomy in autosomal dominant polycystic kidney disease. J Endourol. 2004;18(4):379-82. doi: <http://dx.doi.org/10.1089/089277904323056942>. PubMed PMID: 38680098.

58. Lee YR, Lee KB. Ablation of symptomatic cysts using absolute ethanol in 11 patients with autosomal-dominant polycystic kidney disease. Korean J Radiol. 2003;4(4):239-42. PubMed PMID: 14726641.

59. Liu Y, Li Y, Li N, Xue D, Liu C, Liu S, et al. Flexible ureteroscopy and holmium laser lithotripsy for treatment of upper urinary tract calculi in patients with autosomal dominant polycystic kidney disease. Urological Research. 2012;40(1):87-91. doi: <http://dx.doi.org/10.1007/s00240-011-0390-x>. PubMed PMID: 51441778.

60. Rehman J, Landman J, Andreoni C, McDougall EM, Clayman RV. Laparoscopic bilateral hand assisted nephrectomy for autosomal dominant polycystic kidney disease: initial experience. J Urol. 2001;166(1):42-7. PubMed PMID: 11435819.

61. Rizk D, Jurkovitz C, Veledar E, Bagby S, Baumgarten DA, Rahbari-Oskoui F, et al. Quality of life in autosomal dominant polycystic kidney disease patients not yet on dialysis. Clin J Am Soc Nephrol. 2009;4(3):560-6. doi: <https://dx.doi.org/10.2215/CJN.02410508>. PubMed PMID: 19261830.

62. Sakuhara Y, Nishio S, Morita K, Abo D, Hasegawa Y, Yuasa N, et al. Transcatheter Arterial Embolization with Ethanol Injection in Symptomatic Patients with Enlarged Polycystic Kidneys. Radiology. 2015;277(1):277-85. doi: <https://dx.doi.org/10.1148/radiol.2015141637>. PubMed PMID: 25923222.

63. Seshadri PA, Poulin EC, Pace D, Schlachta CM, Cadeddu MO, Mamazza J. Transperitoneal laparoscopic nephrectomy for giant polycystic kidneys: a case control study. Urology. 2001;58(1):23-7. PubMed PMID: 11445473.

64. Struthers NW, Goldstein MB, Campbell JP, Common AA. Percutaneous aspiration of polycystic kidneys. Minimally Invasive Therapy and Allied Technologies. 1997;6(4):349-51. doi: <http://dx.doi.org/10.3109/13645709709153088>. PubMed PMID: 27474728.

65. Sulikowski T, Kaminski M, Rozanski J, Zietek Z, Domanski L, Majewski W, et al. Laparoscopic removal of renal cysts in patients with ADPKD as an alternative method of treatment and patient preparation for kidney transplantation: preliminary results. Transplant Proc. 2006;38(1):23-7. PubMed PMID: 16504654.

66. Sulikowski T, Tejchman K, Zietek Z, Rozanski J, Domanski L, Kaminski M, et al. Experience with autosomal dominant polycystic kidney disease in patients before and after renal transplantation: a 7-year observation. Transplant Proc. 2009;41(1):177-80. doi: <https://dx.doi.org/10.1016/j.transproceed.2008.10.034>. PubMed PMID: 19249508.

67. Suwabe T, Ubara Y, Sekine A, Ueno T, Yamanouchi M, Hayami N, et al. Effect of renal transcatheter arterial embolization on quality of life in patients with autosomal dominant polycystic kidney disease. Nephrol Dial Transplant. 2017;32(7):1176-83. doi: <https://dx.doi.org/10.1093/ndt/gfx186>. PubMed PMID: 28873973.

68. Christophe JL, Van Ypersele De Strihou C, Pirson Y, Jadoul M, Goffin E, Bernis P, et al. Complications of autosomal dominant polycystic kidney disease in 50 haemodialysed patients. A case-control study. Nephrol Dial Transplant. 1996;11(7):1271-6. doi: <http://dx.doi.org/10.1093/ndt/11.7.1271>.

69. Fitzpatrick PM, Torres VE, Charboneau JW, Offord KP, Holley KE, Zincke H. Long-term outcome of renal transplantation in autosomal dominant polycystic kidney disease. Am J Kidney Dis. 1990;15(6):535-43. PubMed PMID: 2195871.

70. Ishikawa I, Chikamoto E, Nakamura M, Asaka M, Tomosugi N, Yuri T. High incidence of common bile duct dilatation in autosomal dominant polycystic kidney disease patients. Am J Kidney Dis. 1996;27(3):321-6. PubMed PMID: 8604699.

71. Jacquet A, Pallet N, Kessler M, Hourmant M, Garrigue V, Rostaing L, et al. Outcomes of renal transplantation in patients with autosomal dominant polycystic kidney disease: a nationwide longitudinal study. Transpl Int. 2011;24(6):582-7. doi: <https://dx.doi.org/10.1111/j.1432-2277.2011.01237.x>. PubMed PMID: 21352383.

72. Lifson BJ, Teichman JM, Hulbert JC. Role and long-term results of laparoscopic decortication in solitary cystic and autosomal dominant polycystic kidney disease. J Urol. 1998;159(3):702-5; discussion 5-6. PubMed PMID: 9474129.

73. Morino M, De Giuli M, Festa V, Garrone C. Laparoscopic management of symptomatic nonparasitic cysts of the liver: Indications and results. Ann Surg. 1994;219(2):157-64. doi: <http://dx.doi.org/10.1097/00000658-199402000-00007>. PubMed PMID: 24064072.

74. Neijenhuis MK, Gevers TJ, Hogan MC, Kamath PS, Wijnands TF, van den Ouweland RC, et al. Development and Validation of a Disease-Specific Questionnaire to Assess Patient-Reported Symptoms in Polycystic Liver Disease. Hepatology. 2016;64(1):151-60. doi: <https://dx.doi.org/10.1002/hep.28545>. PubMed PMID: 26970415.

75. Timio M, Monarca C, Pede S, Gentili S, Verdura C, Lolli S. The spectrum of cardiovascular abnormalities in autosomal dominant polycystic kidney disease: a 10-year follow-up in a five-generation kindred. Clin Nephrol. 1992;37(5):245-51. PubMed PMID: 1606775.

76. D'Agnolo HMA, Casteleijn NF, De Fijter HW, Messchendorp LA, Peters DJ, Salih M, et al. The association of combined total kidney and liver volume with gastrointestinal symptoms and pain in patients with later stage ADPKD. Nephrol Dial Transplant. 2016;1):i92. doi: <http://dx.doi.org/10.1093/ndt/gfw156.9>. PubMed PMID: 72326120.

77. D'Agnolo HMA, Casteleijn NF, Gevers TJG, de Fijter H, van Gastel MDA, Messchendorp AL, et al. The Association of Combined Total Kidney and Liver Volume with Pain and Gastrointestinal Symptoms in Patients with Later Stage Autosomal Dominant Polycystic Kidney Disease. Am J Nephrol. 2017;46(3):239-48. doi: <https://dx.doi.org/10.1159/000479436>. PubMed PMID: 28881341.

78. Eriksson D, Karlsson L, Eklund O, Dieperink H, Honkanen E, Melin J, et al. Health-related quality of life across all stages of autosomal dominant polycystic kidney disease. Nephrol Dial Transplant. 2017;32(12):2106-11. doi: <https://dx.doi.org/10.1093/ndt/gfw335>. PubMed PMID: 27662885.

79. Hogan MC, Abebe K, Torres VE, Chapman AB, Bae KT, Tao C, et al. Liver involvement in early autosomal-dominant polycystic kidney disease. Clin Gastroenterol Hepatol. 2015;13(1):155-64.e6. doi: <https://dx.doi.org/10.1016/j.cgh.2014.07.051>. PubMed PMID: 25111236.

80. Miskulin DC, Abebe KZ, Chapman AB, Perrone RD, Steinman TI, Torres VE, et al. Health-related quality of life in patients with autosomal dominant polycystic kidney disease and CKD stages 1-4: A cross-sectional study. Am J Kidney Dis. 2014;63(2):214-26. doi: <http://dx.doi.org/10.1053/j.ajkd.2013.08.017>. PubMed PMID: 52851109.

81. Suwabe T, Ubara Y, Mise K, Kawada M, Hamanoue S, Sumida K, et al. Quality of life of patients with ADPKD-Toranomon PKD QOL study: cross-sectional study. BMC Nephrol. 2013;14:179. doi: <https://dx.doi.org/10.1186/1471-2369-14-179>. PubMed PMID: 23978051.

82. Taylor M, Johnson AM, Tison M, Fain P, Schrier RW. Earlier diagnosis of autosomal dominant polycystic kidney disease: importance of family history and implications for cardiovascular and renal complications. Am J Kidney Dis. 2005;46(3):415-23. PubMed PMID: 16129202.
