# Supplementary material for: Patient-reported outcome measures for pain in autosomal dominant polycystic kidney disease: A systematic review
Source: PLoS One. 2021 May 27;16(5):e0252479. doi: 10.1371/journal.pone.0252479 (PMC8158964; doi:10.1371/journal.pone.0252479)
Supplement: S4 Table — (DOCX) [file pone.0252479.s004.docx]

**S4 Table. Psychometric properties of measures that have been used to assess pain in patients with ADPKD**

| **Measure** | **Validity** | **Reliability** | | |
| --- | --- | --- | --- | --- |
| **ADPKD-IS[1]** | Content: measure development began with a literature review, clinician interviews and focus groups with patients, to generate concepts which informed the creation of a draft measure. Following cognitive interviews and focus groups with patients, the measure was refined to ensure it was comprehensible, fully captured the patient experience and interpreted consistently across cultural groups. The final measure was then reviewed and endorsed by a team of clinical experts.  Construct: confirmatory factor analysis did not support the initially hypothesized two-factor structure of the measure, but instead a three-factor structure including four domain-independent items which were ultimately retained due to patient and clinician endorsement. Item discrimination for all items were above the recommended criterion of 0.80, there was adequate homogeneity of variance in each domain (<3.0), and item-total correlations confirmed all items were sufficiently correlated with the domains they were hypothesized to be part of against a criterion of 0.40.  *Convergent:* N/A  *Discriminant:* changes in the physical domain score between baseline and follow-up were correlated with changes in pain intensity as measured by Global rating of change in pain scale the pain intensity domain of the BPI-SF. Changes from baseline in fatigue and emotion domain scores were not correlated with changes in pain intensity, and none of the domain change scores were correlated with changes in SF-12 Physical Component Summary (PCS) or Mental Component Summary (MCS) scores. Scores on the physical, emotional and fatigue domains of the ADPKD-IS were significantly higher for patients in CKD stage 3b-5 compared to patients in CKD stage 1. Percentage difference between CKD stage 1 and later stages was more pronounced for the ADPKD-IS compared to the SF-12 PCS/MCS and EQ-5D.  Criterion:  *Predictive:* N/A  *Concurrent:* correlations of domains with the SF-12 summary scores were: –0.68 for physical domain with the PCS, –0.58 for fatigue domain with the PCS, and –0.54 for emotional domain with the MCS. This indicated sufficient convergent validity. | | Test-retest: coefficients were 0.89 for physical domain, 0.92 for fatigue domain, and 0.86 for emotional domain. In addition to high Intraclass correlations between time points at 3 and 4 weeks, it suggested a high level of test-retest reliability.  Internal consistency: the measure demonstrated adequate internal consistency with Cronbach’s alphas of 0.94 for physical domain, 0.94 for fatigue domain, and 0.85 for emotional domain. Inter-item correlations of 0.71 for physical domain, 0.84 for fatigue domain, and 0.58 for emotional domain similarly indicated adequate internal consistency between domain items. |  |
| **PLD-Q[2]**  *46% Dutch and 82% US patients* | Content: measure was created through a process of literature search and interviews with patients and clinicians, followed by a first validation study in the Netherlands (NL). The measure was then amended and improved through cognitive interviews and focus groups with patients and clinicians, before a subsequent validation study in the United States (US).  Construct: factor analysis supported a unidimensional structure of the PLD-Q (Factor 1 eigenvalues= 6.15 [Dutch] and 8.57 [US]).  *Convergent:* N/A  *Discriminant:* negative correlation between PLD-Q total score and global health VAS (NL, 0.517; US, 0.599; P< 0.001). ADPKD patients without PLD scored lower than PLD patients from the US *(*P*<* 0.001). All PLD patients and ADPKD patients (without PLD) from the US scored significantly higher on PLD-Q than healthy controls (P< 0.001, P= 0.006 respectively).  Criterion:  *Predictive:* N/A  *Concurrent:* positive correlation between PLD-Q total score and EORTC symptom burden score (NL, 0.788; US, 0.811; P< 0.001). | | Test-retest: test-retest on a subsample of participants were excellent (ICC 0.94, 95% CI 0.88-0.97 for Dutch and 0.96, 95% CI 0.94‐0.97 for US).  Internal consistency: Cronbach’s alpha in the Dutch and US groups was 0.796 and 0.840 respectively, indicating high internal consistency. |  |

Note: Validation studies were excluded if they were not available in full, were for a translation of the original measure or were not written in English.

**References**

1. Oberdhan D, Cole J,  Krasa HB, Cheng R, Czerwiec FS, Hays RD, et al. Development of the Autosomal Dominant Polycystic Kidney Disease Impact Scale: A New Health-Related Quality-of-Life Instrument. Am J Kidney Dis. 2018;71(2):225-35. doi: doi: 10.1053/j.ajkd.2017.08.020.

2. Neijenhuis MK, Gevers TJ, Hogan MC, Kamath PS, Wijnands TF, van den Ouweland RC, et al. Development and Validation of a Disease-Specific Questionnaire to Assess Patient-Reported Symptoms in Polycystic Liver Disease. Hepatology. 2016;64(1):151-60. doi: <https://dx.doi.org/10.1002/hep.28545>. PubMed PMID: 26970415.
