# Supplementary material for: Patient-reported outcome measures for pain in autosomal dominant polycystic kidney disease: A systematic review
Source: PLoS One. 2021 May 27;16(5):e0252479. doi: 10.1371/journal.pone.0252479 (PMC8158964; doi:10.1371/journal.pone.0252479)
Supplement: S5 Table — (DOCX) [file pone.0252479.s005.docx]

**S5 Table. Dimensions of pain assessed by measures used in patients with ADPKD**

| **Dimension** | **Definition** | **Example of item** |
| --- | --- | --- |
| **Site dimensions** |  |  |
| Abdomen – General | Experiencing pain in the abdominal region | Over the past weeks, how often have you experienced abdominal pain? |
| Abdomen – Upper abdomen | Experiencing pain in the upper abdomen pain (e.g. stomach aches or liver pain) | Over the past weeks, how often have you experienced upper abdomen pain? |
| Abdomen – Lower abdomen | Experiencing pain in the lower abdomen (e.g. kidney pain) | Over the past weeks, how often have you experienced lower abdomen pain? |
| Abdomen – Flank | Experiencing pain in the side | Over the past weeks, how often have you experienced flank pain? |
| Lower back | Experiencing lower back pain | Over the past weeks, how often have you experienced back pain? |
| Thorax – Chest | Experiencing chest pain (e.g. angina pectoris, myocardial infarction or pericarditis) | Over the past weeks, how often have you experienced chest pain? |
| Thorax – Rib cage | Experiencing pain or pressure in the rib cage | Over the past weeks, how often have you experienced pain or pressure in the rib cage? |
| Head and Face | Experiencing acute or chronic tension headache | Over the past weeks, how often have you experienced headache? |
| Generalized body | Experiencing bodily or generalized pain | Over the past weeks, how often have you experienced bodily pain? |
| Non-specified | Experiencing other pain | Over the past weeks, how often have you experienced pain? |
| **Measurement dimensions** |  |  |
| Intensity | The degree of overall pain felt by patient | How intense is the pain which you have been experiencing? |
| Frequency | Number of times patient has felt pain in a given time frame | Over the past weeks, how often have you experienced pain? |
| Temporality | Difference in pain felt by the patient in a given time frame | To what degree has your pain changed during the past week? |
| Sensory | Different sensation of pain felt by patient | Over the past weeks, can you describe which sensation of pain have you experienced? |
| **Type dimensions** | | |
| Nociceptive pain – Visceral | Injury/damage to internal organs internal organs (liver, kidney), abdomen, chest, epigastric soreness, heartburn | I have back/flank/abdominal pain / I have chest pain |
| Nociceptive pain – Somatic | Injury/damage to muscle, rib cage, headaches, sprains and cramps | I have muscles aches/cramps / I have headaches |
| Neuropathic pain | Experiencing a not-burning or cold-freezing pain, pain caused by light touch, itching, tingling or pins and needles, or numbness | I have not-burning pain / I have cold-freezing pain |
| **Impact dimensions** |  |  |
| Life participation – Daily activity | The impact of pain on the ability to participate in life activities (e.g. general and daily activities) | Because of pain, I have to limit my daily activities |
| Life participation – Social activity | The impact of pain on the ability to participate in life activities (e.g. social activities and hobbies) | Because of pain, I have to limit my social activities |
| Life participation – Work | The impact of pain on the ability to participate in life activities (e.g. work outside the home or housework) | Because of pain, I have to limit my work or housework |
| Life participation – Walking ability | The impact of pain on the ability to participate in life activities (e.g. walking ability) | Because of pain, I have to limit my walking abilities |
| Life participation – Physical function | The impact of pain on the ability to participate in life activities (e.g. daily chores, commitments) | Because of pain, I have to limit my daily exercises |
| Life participation – Strenuous physical activity | The impact of pain on the ability to participate in strenuous physical activity | Because of pain, I have to limit my strenuous physical activities |
| Sleep | The impact of pain on sleep (e.g. sleep quality, latency and duration) | I have trouble to sleep |
| Mental – Mood | Feeling anxious, depressed, apathetic or irritable before, during and after pain | Did pain interfere with your mood? |
| Mental – Bother | Feeling bothered before, during and after pain | How pain bother you? |
| Mental – Anxiety | Feeling anxious and depressed before, during and after pain | Did pain make you anxious? |
| Mental – Affective | Feeling tired, exhausted, sickening, fearful and punishing-cruel before, during and after pain | Did pain make you tired, exhausted, sickened, or fearful? |
| Mental – Enjoyment of life | Feeling tired, anxious, depressed, apathetic or irritable before, during and after pain | Did pain interfere with your enjoyment of life? |
